# A hybrid PSO–FPA metaheuristic algorithm for ultra-low sidelobe and high-directivity synthesis of concentric circular antenna arrays for advanced radar applications

**DOI:** 10.1038/s41598-026-36315-6

**Published:** 2026-02-03

**Authors:** Mohammed Brahimi, Imane Haouam, Riyadh Bouddou, Omar Almomani, Ayodeji Olalekan Salau, Iryna Hunko

**Affiliations:** 1 Department of Electrical Engineering Faculty of Technology , University of Naama , 45000 Naama, Algeria; 2 Department of Networks and Cybersecurity, Hourani Center for Applied Scientific Research, Al-Ahliyya Amman University, Amman, Jordan; 3 Department of Electrical/Electronics and Computer Engineering , Afe Babalola University , Ado-Ekiti, Nigeria; 4 Saveetha School of Engineering , Saveetha Institute of Medical and Technical Sciences , Chennai, Tamil Nadu India; 5 Department of Electric Station and System , Vinnytsia National Technical University , 95 Khmelnytske shose, Vinnytsia, 21021 Ukraine

**Keywords:** Antenna array synthesis, Beamforming, Concentric circular antenna array, Directivity enhancement, Flower pollination algorithm, Metaheuristic optimization, Particle swarm optimization, Sidelobe level reduction, Engineering, Mathematics and computing

## Abstract

This paper introduces a novel hybrid PSO–FPA metaheuristic algorithm that integrates the global exploration capability of the Flower Pollination Algorithm (FPA) with the adaptive convergence and dynamic search behavior of Particle Swarm Optimization (PSO) for the efficient synthesis of Concentric Circular Antenna Arrays (CCAAs). By embedding PSO’s inertia-weighted velocity update and acceleration coefficients into FPA’s global and local pollination phases, the proposed approach establishes a self-adaptive optimization framework capable of achieving an effective trade-off between global exploration and local exploitation. The algorithm is applied to the simultaneous optimization of excitation amplitudes and ring radii under four design configurations, considering both with and without central element scenarios. The optimization objective focuses on minimizing Side Lobe Levels (SLL) while maintaining high directivity and narrow Half-Power Beamwidth (HPBW). Comprehensive numerical simulations demonstrate that the proposed hybrid PSO–FPA algorithm outperforms conventional metaheuristics—including FPA, PSO, Artificial Bee Colony (ABC), and Whale Optimization Algorithm (WOA)—in terms of sidelobe suppression, convergence speed, and pattern symmetry. The hybrid method achieves a minimum SLL of − 45.01 dB, representing an improvement of approximately 38–42% over traditional techniques, and enhances beam symmetry and directivity by 24–28%, achieving up to 13.14 dB of main-lobe gain with minimal beamwidth degradation. Moreover, the joint optimization of amplitudes and ring radii yields a balanced radiation performance, characterized by focused beams with sidelobes below − 45 dB and computation times under 12 s per design. The results confirm that the proposed PSO–FPA metaheuristic delivers superior sidelobe suppression, enhanced beam control, and rapid convergence, making it a robust and scalable optimization tool for next-generation antenna synthesis in radar, wireless communication, and smart sensing systems requiring precise directional control and interference mitigation.

## Introduction

In the ever-growing domain of wireless and mobile communications, requirements for the development of agile and intelligent antenna systems become urgent. Due to the currently increasing demands related to higher data rates, broader coverage, and adaptive beam control, antenna arrays have managed to emerge as critical building blocks for modern communication architectures. At the heart of their functionality lies an elaborate but also essential task, that of array synthesis, where spatial configurations and excitation profiles are laboriously tailored to achieve optimal radiation performance. The geometrical and excitation structure constitute the most fundamental determinants of the form and function of an antenna array. Ranging from linear to circular, concentric, and rectangular arrangements, each of these topologies offers a unique blend of directivity, azimuthal coverage, and complexity^1–3^. While linear arrays have been valued for their simplicity in design and very good directivity, they are limited by symmetrical major lobes that waste energy in undesired backward directions. Such limitations restrict their efficiency in full-azimuth applications^4,5^. To overcome these angular limitations, researchers turned to circular arrays because their rotational symmetry offers uniform coverage and allows electronic beam steering without any physical variation in structure. The price paid in this design was elevated Side Lobe Levels (SLLs), which may cause interference and signal deterioration. With a view to overcoming this challenge, one introduced the CCAA geometry that integrates multiple circular rings of different radii and nonuniform element distributions around a common center^6^. Besides symmetrical beam shaping, such geometry allows for enhanced spatial coverage and hence finds excellent applications in direction finding, broadband beamforming, and smart sensing systems^7^. Despite such promising architecture, the optimization of excitation currents and element positions in CCAAs is a computationally intensive task. The traditional deterministic approaches to optimization often cannot provide satisfactory results regarding nonlinear, multimodal objective functions and usually converge to suboptimal local minima or fail to scale with the dimensionality of the design space. Bio-inspired optimization algorithms are today increasingly popular due to their adaptiveness and capability for global search. In this regard, Particle Swarm Optimization (PSO) has become one of the landmark methods. Based on the behavioral dynamics of bird flocking and fish schooling, PSO provides a population-based paradigm wherein one can efficiently explore complex design spaces without the associated computational burden of gradient-based methods^8–10^.

For the optimisation of complex electromagnetic structures like antenna arrays, gradient-based methods are often hindered by nonlinearities, multimodality, and high dimensionality. In contrast, population-based metaheuristics, such as Particle Swarm Optimization (PSO), excel at exploring such intricate design spaces without requiring gradient information, thus avoiding associated computational burdens and convergence to local minima. The application of PSO in electromagnetic design remains an active and fruitful research area, as evidenced by its recent and successful deployment in diverse domains: from the synthesis of time-modulated arrays^11^ and electronic design automation for interconnect modelling^12^, to the optimisation of microwave components^13^, semiconductor devices^14,15^, and advanced packaging solutions^16^. This breadth of applications underscores PSO’s versatility and robustness as a global optimiser.

Its flexibility and convergence efficiency have positioned it as one of the preferred tools in electromagnetics and antenna array synthesis^17^. The progress on metaheuristics during the last three decades has resulted in a variety of nature-inspired algorithms. The relevant contributions include the Genetic Algorithm (GA)^18–20^, EP^21^, and Biogeography-Based Optimization (BBO): Dib et al. managed to successfully use it for non-uniform CCAA design^22^. Another representative example is the Firefly Algorithm (FA), the swarm luminance behavior of fireflies, which was implemented^23^ for effective synthesis of circular arrays. Most recently, the Symbiotic Organisms Search (SOS) algorithm, which constitutes an ecological optimization approach inspired by mutualistic and competitive relationships occurring in nature, was proposed for SLL suppression in CCAA configurations^24^. Researchers applied another representative example, the Backtracking Search Optimisation (BSA) algorithm, a population-based evolutionary method with simple control parameters, for low-SLL CCAA synthesis at a fixed beamwidth^25^. Opposition-based BAT (OBAT) is proposed, which enhanced standard BAT echolocation with opposition-based learning for faster convergence, and was applied to circular concentric arrays^26^. The Dragonfly Algorithm (DA), a nature-inspired method based on static/dynamic swarming behaviours, was later proposed by Babayigit CCAAs^27^, achieving low SLL. The Political Optimiser (PO), a socio-inspired method for modelling multi-stage political processes, outperformed both FA and SOS in three-ring CCAAs by optimising amplitudes and spacings to reach lower SLLs^28^.

### Motivation and objectives

The rapid growth of algorithmic paradigms in computational optimization has favored the synthesis of hybrid techniques that make effective use of the complementary strengths of multiple strategies. For the present study, a novel hybrid optimization framework has been developed, which integrates the global search capabilities of PSO with the localized intensification mechanisms of Flower Pollination Algorithm (FPA). Based on the natural process of reproduction of flowering plants and the pollinator behavior, FPA, which is proposed, represents a very powerful approach to refine solutions locally and maintain a global diversity^29^. Incorporating the swarm intelligence-driven exploration capability of PSO with the pollination-inspired exploitation of FPA, the hybrid algorithm intends to obtain a well-balanced trade-off between global convergence and local refinement. This can facilitate the solving of the complex, multimodal antenna design problems where high-fidelity tuning has to be performed together with the capability of global adaptation.

The synthesis of CCAA radiation patterns with constrained sidelobes constitutes a complex, non-convex, and multimodal optimisation problem in a high-dimensional parameter space. For such problems, derivative-free, population-based metaheuristics offer a powerful and direct solution strategy. While state-of-the-art machine learning (ML) approaches, particularly deep learning, excel at pattern recognition and building fast surrogate models from large datasets, they require substantial pre-computed data for training, and their optimality is inherently linked to the quality and scope of that training set. In contrast, metaheuristics like PSO and FPA are direct optimizers; they navigate the objective landscape without requiring gradient information or a pre-existing dataset and seek the global optimum through iterative exploration and exploitation. This advantage makes them particularly suited for original design synthesis, where the goal is to discover novel, high-performance configurations, not to replicate or interpolate from known examples. The choice to hybridise PSO and FPA is motivated by their complementary strengths in balancing intensive local search (PSO) and broad global exploration (FPA), creating a robust solver tailored for the challenging, ultra-low sidelobe objectives of this work.

In order to validate the effectiveness of the proposed hybrid method, it has been applied to the synthesis of CCAA under two different design scenarios: one excluding the central element and the other including it. The optimization objective aims at the minimization of SLLs while maintaining desirable beamwidth characteristics. It turns out that the inclusion of the central component significantly reduces SLLs with just a marginal increment of beamwidth, an advantageous compromise for high-resolution radar and advanced communication systems. Accordingly, the key objectives of the work are as follows:

- To develop and implement a hybrid PSO-FPA algorithm suited for CCAA synthesis.
- To assess the algorithm’s performance when it comes to optimizing antenna parameters that are under varying configuration constraints.
- To study the trade-offs between SLL suppression and variation in beamwidth, with a view to the eventual incorporation of a central element.
- To provide insight into the algorithm’s convergence behavior and its practical applicability for high-performance antenna systems.

The rest of the paper is organized as follows: section "Mathematical modelling" provides the mathematical formulation, cost function design for CCAA synthesis, brief overviews of PSO and FPA methodologies, and the proposed hybrid algorithm. The section "Numerical results and discussion" presents the results obtained from the simulation studies. The section "Comparative study" addresses a comparative study. Finally, the section "Conclusions" concludes the study and provides guidelines for the future scope.

## Mathematical modelling

### Geometrical design and spatial arrangement of the CCAA

**Fig. 1:**
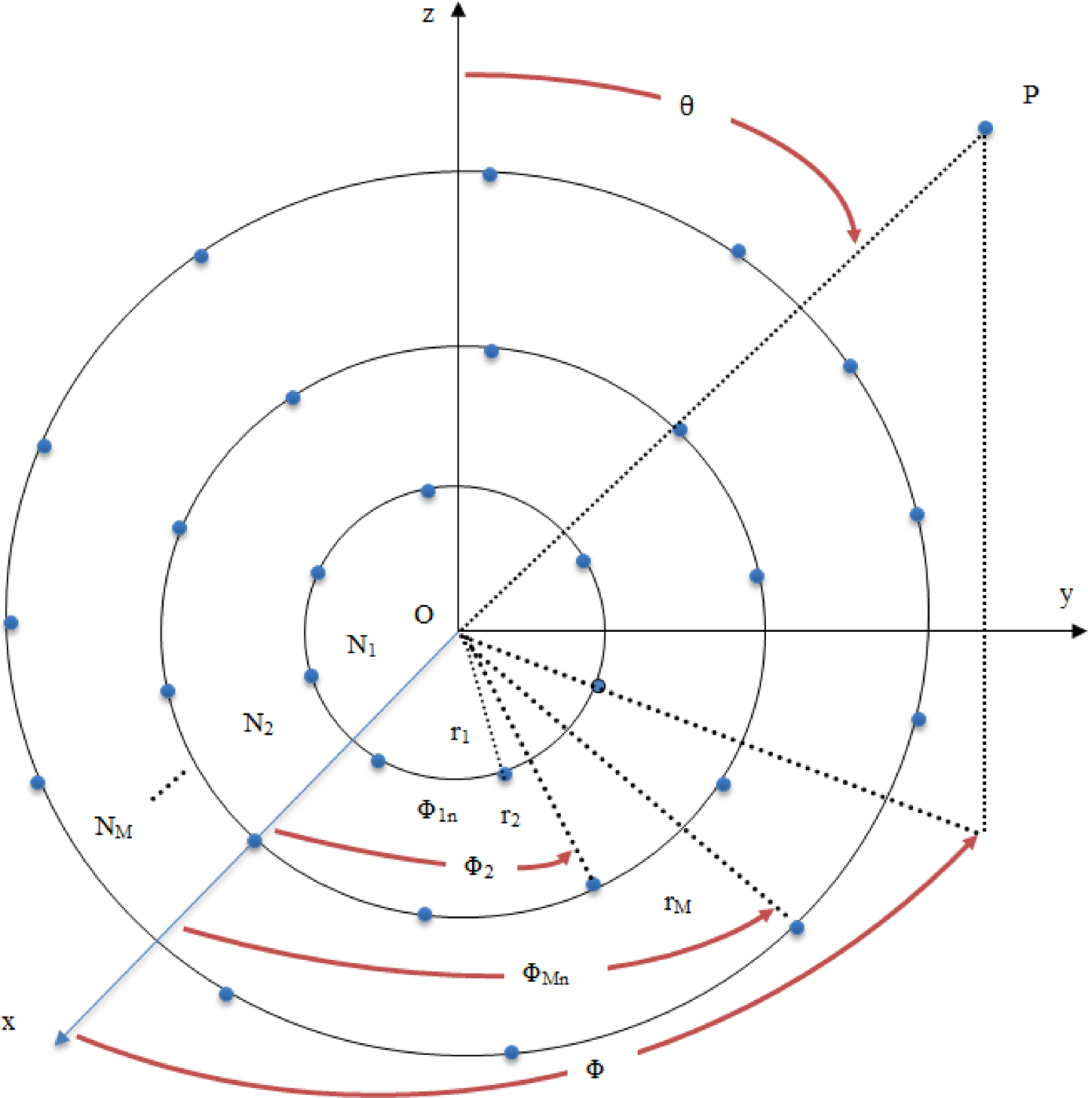
Typical configuration of a CCAA with multiple rings and element distribution.

The CCAA architecture encompasses a set of multiple concentric rings, each hosting a uniformly distributed set of radiating elements. Indeed, this geometry provides natural rotational symmetry and better beamforming capabilities in azimuthal scanning. Let the array be made up of M concentric rings; the radius of the $\:m$-th ring is$\:{\:r}_{m}$, which can accommodate $\:{N}_{m}$ equally spaced antenna elements. This is illustrated in Fig. 1, where this ring-based structure naturally allows for an elegant mathematical representation of the Array Factor (AF) that governs the resulting radiation pattern. The AF is indeed a key descriptor of the radiation pattern and represents the spatial summation of all the individual contributions from these elements. It is a function of the observation direction defined by zenith angle θ and azimuth angle ϕ, and is mathematically expressed as^21,30^:

1 $$\:AF\left(\theta\:,\varphi\:\right)=\sum\:_{m=1}^{M}\sum\:_{i=1}^{{N}_{m}}{W}_{m}{e}^{j\left[{K.r}_{m}.\mathrm{sin}\left(\theta\:\right).\mathrm{cos}\left(\varphi\:-{\varphi\:}_{mn}+{\alpha\:}_{mn}\right)\right]}$$

Here, $\:{W}_{m}$ is the excitation amplitude for elements on the $\:m$-th ring, $\:{\varphi\:}_{mn}$ is the angular position of the $\:n$-th element, $\:{\alpha\:}_{mn}$ is the steering phase shift, and $\:K$ is the wave number.

To ensure uniform angular distribution along each ring, the angular position $\:{\varphi\:}_{mn}$ of the $\:n$-th element on the $\:m$-th ring is determined by^21^:

2 $$\:{\varphi\:}_{mn}=2\pi\:\frac{\left(n-1\right)}{{N}_{m}}$$

This evenly spaced placement allows the antenna array to take on a symmetric configuration, which is important for predictable radiation behavior and for a simplified analytical model. In order to steer the beam in the desired direction specified by the angles $\:({\theta\:}_{0},\:\:{\varphi\:}_{0})$, a steering phase, $\:{\alpha\:}_{mn}$ is introduced. This phase shift compensates for the spatial delay across the array and is given by:

3 $$\:{\alpha\:}_{mn}=-{K.r}_{m}.\mathrm{sin}\left({\theta\:}_{0}\right).\mathrm{cos}\left({\varphi\:}_{0}-{\varphi\:}_{mn}\right)$$

This formulation ensures coherent wavefront alignment in the steering direction, so that precise main lobe control is achievable without degradation of beam shape or sidelobe symmetry. For comparison and visualization purposes, the magnitude of the AF is usually normalized and expressed in decibels (dB). The normalized AF is given as:

4 $$\:AF\left(\theta\:,\varphi\:\right)=20{\mathrm{log}}_{10}\left[\frac{\left|AF\left(\theta\:,\varphi\:\right)\right|}{\left|AF{\left(\theta\:,\varphi\:\right)}_{max}\right|}\right]$$

This logarithmic representation highlights the relative strengths of sidelobes and facilitates quantitative assessments of the radiation pattern’s quality.

### Cost function formulation

In order to effectively suppress the maximum SLL by the definition of the CCAA, an appropriate Cost Function (CF), also called a fitness function, needs to be designed. This should focus on the minimization of the maximum SLL while preserving a HPBW comparable with that of a uniformly excited antenna array. With this in mind, the CF is defined as:

5 $$\:CF={W}_{1}.{SLL}_{max}+{W}_{2}\left(FNB{W}_{c}-FNB{W}_{u}\right)$$

In this formulation, $\:{SLL}_{max}$ represents the magnitude of the maximum side-lobe level. Meanwhile, $\:FNB{W}_{c}$ denotes the first null beamwidth in radians for the non-uniform and uniform excitations, respectively. $\:{W}_{m}\:$is the excitation current of the $\:m$-th ring element, and for the case of uniform excitation, where uniform excitation implies $\:{W}_{m}=1$ for all elements? The weighting coefficients $\:{W}_{1}$ and $\:{W}_{2}$ play a crucial role in balancing the influence of the two objectives. In general, the larger the relative weighting, the stronger the emphasis the formulation will place on side-lobe suppression relative to beamwidth matching. In this respect, experimental investigations have identified that $\:{W}_{1}=1$ and $\:{W}_{2}=1$ provide the most favorable outcome. This ensures that the reduction of $\:{SLL}_{max}$ is significantly larger than any minor variation in beamwidth and also maintains the CF as non-negative. Through minimizing the CF of Eq. (5), the design process is assured of achieving a twofold objective: a significant reduction in the maximum SLL and a minimum discrepancy between the first null beamwidths of the nonuniform and uniform excitation configurations. The resulting antenna array design is therefore more effective and optimized for high-performance applications.

### Fundamentals of PSO algorithm

In PSO^31,32^, each particle represents a potential solution, characterized by three D-dimensional vectors: the current position $\:{x}_{i}=\left[{x}_{i}^{1},{x}_{i}^{2},\dots\:,{x}_{i}^{D}\right]$, the personal best position $\:{p}_{i}=\left[{p}_{i}^{1},{p}_{i}^{2},\dots\:,{p}_{i}^{D}\right]$, and the velocity $\:{v}_{i}=\left[{v}_{i}^{1},{v}_{i}^{2},\dots\:,{\:v}_{i}^{D}\right]$. The individual best ($\:{p}_{i}$) is the best position the particle has discovered so far based on the fitness function, while the global best ($\:{p}_{g}=[{{p}_{g}}^{1},{pg}^{2},\dots\:,{{p}_{g}}^{D}]$) represents the best position identified by the entire swarm. The dynamics of particle movement are governed by iterative updates to both velocity and position, as defined by the following equations:

6 $$\:{v}_{i}^{d}\left(k+1\right)=\omega\:{v}_{i}^{d}\left(k\right)+{C}_{1}{r}_{1}^{d}\left({p}_{i}^{d}\left(k\right)-{x}_{i}^{d}\left(k\right)\right)+{C}_{2}{r}_{2}^{d}\left({{p}_{g}}_{i}^{d}\left(k\right)-{x}_{i}^{d}\left(k\right)\right)$$

7 $$\:{v}_{i}^{d}\left(k+1\right)={v}_{i}^{d}\left(k+1\right)+{x}_{i}^{d}\left(k\right)$$

The parameter ω indicates the inertia weight, which controls the trade-off between global exploration and local exploitation of the search space. Parameters C_1 and C_2 are the cognitive and social acceleration coefficients, respectively, controlling how much the particle is attracted to its previously best position or to the global best. Variables r_1^d and r_2^d are random numbers uniformly distributed in the range [0, 1], which introduce stochastic behavior for the sake of search diversity. A particle dynamically adjusts its flying velocity through integrating three components: inertia (the previous velocity), cognitive learning (distance from personal best), and social learning (distance from global best). Position update follows afterwards. In each cycle, the current fitness of a particle is evaluated, and accordingly, its personal best is updated if a better solution is obtained, which may further update the swarm’s global best in turn. It is crucial that ω balances exploration (global search) and exploitation (local search) and helps the algorithm avoid premature convergence while ensuring robustness in the search behavior of the method^33^. PSO conducts an efficient tour through the fitness landscape in pursuit of the best solution by continuously guiding the particles toward their own experience and the collective experience of the swarm.

### The flower pollination algorithm

The FPA mimics the natural pollination process in flowering plants, where pollination occurs through either biotic agents (e.g., insects and birds) or abiotic factors (e.g., wind and diffusion)^34^. The biotic pollination, which involves long-distance transfer of pollen, can be related to global pollination, while the local pollination corresponds to nearby interactions^35,36^. Each pollen grain represents a solution $\:{X}_{i}$, and the whole flower population corresponds to the solution space. The process of global pollination is modeled through Lévy flights, allowing exploration through large steps, and can mathematically be written as^37^:

8 $$\:{X}_{i}^{\left(t+1\right)}={X}_{i}^{t}+\gamma\:L\left(y\right)\left({X}_{i}^{t}-G\right)$$

Where$\:\:\gamma\:$is the scaling factor, $\:G$ is the current best global solution, and $\:L\left(y\right)$ is the pollination step size drawn from the Lévy distribution:

9 $$\:L\approx\:\frac{\lambda\:\varGamma\:\left(\lambda\:\right)\mathrm{sin}\left(\pi\:\frac{\lambda\:}{2}\right)}{\pi\:.{S}^{1+\lambda\:}},\lambda\:=1.5$$

Local pollination simulates short-range solution refinement and is defined by:

10 $$\:{X}_{i}^{\left(t+1\right)}={X}_{i}^{t}+\epsilon\:\left({X}_{l}^{t}-{X}_{k}^{t}\right)$$

Where $\:{X}_{l}^{t}\:$and $\:{X}_{k}^{t}$ are pollen from different flowers on the same plant, and $\:\epsilon\:$ is a random number from a uniform distribution in [0, 1]^38,39^.

### The suggested algorithm

In refining nature’s own rituals of optimization, the FPA in its primitive form was no longer sufficient. It needed to evolve further, sharpen its instinct, widen its vision, and stir into an action that would be more purpose-oriented. It was thus that a new algorithm was born, entwining the elegance of natural flower pollination with the precision of PSO. Central to this was a subtle, yet powerful concept-balance. Within the process of global cross-pollination, embedding the concept of inertial weight from PSO particles within disciplined flight paths taught the algorithm to dance precariously between the wild, unrestrained wanderings of exploration and the focused determination of exploitation. It is no longer a directionless wanderer; it is now a seeker with intent, homing in on solutions with increased precision. But that was not all. The self-pollination process, once a humble, local affair, has been reimagined with newfound vigor. It was stitched with two acceleration coefficients adapted from PSO heritage. Like twin engines, these propelled the algorithm past snares of local optima, gave it the resilience to press through deceptively large valleys, and made for higher promises of peaks. Global pollination, earlier diffuse and without direction, was refined. This became a strategic explorer, dispatched to map faraway lands in the vast search space, ensuring the algorithm would not be couched by the mirage of proximal minima. Simultaneously, local pollination was honed to become a swift, efficient closer in acceleration, speeding up convergence with the urgency of a bee returning toward a familiar bloom. The new rule governing global cross-pollination was articulated as:

11 $$\:{X}_{i}^{\left(t+1\right)}={{C}_{1}X}_{i}^{t}+{C}_{2}L\left({X}_{l}^{t}-{g}^{*}\right)$$

While here, every new location was no longer the result of random flights but an actual path carved out of learned momentum and guiding influence. Meanwhile, self-pollination embraced its new form:

12 $$\:{X}_{i}^{\left(t+1\right)}={X}_{i}^{t}+\omega\:\left({X}_{l}^{t}-{X}_{k}^{t}\right)$$

With this, the algorithm now had inertia with precision, calculated movements, and weighted choices. It would learn from its peers and adjust course with each discovery, refining a strategy with every iteration. Table 1 outlines the procedural flow for the proposed algorithm:

**Table 1:** The pseudocode of the PSO–FPA metaheuristic Algorithm.

Algorithm 1	PSO–FPA Metaheuristic Algorithm
**(1) Start** **(2) Define the Objective Function** Let$\:f\left({x}_{1},{x}_{2},\dots\:,{\:x}_{D}\right)$represent the objective function to be optimized. **(3) Initialization** - Initialize the algorithm parameters. - Set the switching probability$\:p$. - Define the maximum number of generations$\:M$. - Determine the initial best solution from the population. - Identify the global best solution ($\:gbest$) from the initial population. **(4) Termination Criteria Check** - Is the current generation$\:t$less than the maximum$\:M$? ♣ Yes: Proceed to Step (5). ♣ No: Proceed to Step (9). **(5) For Each Flower in the Population (i)** - Check if the current generation$\:t$is less than the maximum allowed generations$\:n$. Yes: Continue; otherwise, increment$\:t$and return to Step 4. **(6) Switching Probability Check** - Generate a random value$\:r$. - If$\:r<p$, perform global pollination using the following update rule: $\:{X}_{i}^{\left(t+1\right)}={{C}_{1}X}_{i}^{t}+{C}_{2}L\left({X}_{l}^{t}-{g}^{*}\right)$ - Else, perform local pollination using the following update rule: $\:{X}_{i}^{\left(t+1\right)}={X}_{i}^{t}+\omega\:\left({X}_{l}^{t}-{X}_{k}^{t}\right)$ **(7) Evaluate New Solutions** - If the newly obtained solution is better than the previous one, update the population with the new solution. **(8) Find Current Best Solution** - After evaluating all flowers in the population, identify the current best solution. **(9) Stop** - Display the best solution obtained throughout the process.	

## Numerical results and discussion

The two significant optimization techniques, namely, PSO and FPA, have been applied for the intensive refinement of the current excitation amplitudes and element spacing of the non-uniform four-ring CCAA array. For the sake of robustness and accuracy, each method has been executed 100 times using MATLAB (R2024) on a system with an Intel(R) Core (TM) i7-6300U CPU at 2.40 GHz and 16 GB of RAM.

The control parameters for the standard PSO and FPA algorithms were selected based on established conventions in their foundational literature and refined through preliminary empirical testing to ensure robust performance for the high-dimensional, non-convex optimisation landscape of CCAA synthesizers.

For PSO, a population of 50 particles provides a balance between search diversity and computational cost for problems with numerous design variables (excitation amplitudes/phases). The cognitive and social coefficients (c1 = c2 = 1.5) follow the common constricted PSO formulation that promotes stable convergence. A constant inertia weight of w = 0.7 offers a persistent exploratory drive, which was found beneficial in preliminary tests to avoid premature stagnation before the FPA’s global search component can be effectively used within the hybrid framework.

For FPA, a switch probability of *p* = 0.8 emphasizes global pollination (Lévy flights), aligning with the need for strong exploration in the initial phases of optimisation. The scaling factor γ = 0.1 controls the step size of local pollination, fine-tuned to prevent overly aggressive local moves. The Lévy exponent β = 1.5 is a standard value that generates step lengths with a heavy-tailed distribution, efficiently spanning the search space. The population size and maximum iteration count were kept consistent with PSO for a fair comparison in the hybrid structure and standalone benchmarks. These parameters were not selected arbitrarily. Initial values were drawn from seminal works^4,42^. Their suitability was then confirmed and marginally tuned through sensitivity analyses on representative CCAA test cases, ensuring they yielded consistent convergence to high-quality solutions. Simulations were performed using carefully chosen control parameters to maximize the performance of the optimization algorithms. The optimal parameter configurations of both algorithms are shown in Table 2. To ensure a fair and unbiased comparison of the proposed PSO-FPA algorithm against the benchmark algorithms (standard PSO, FPA, ABC, and WOA), a strict protocol of identical experimental conditions was adhered to:

Population Size: All algorithms utilised an identical population size of 50 individuals.

Stopping Criterion: Each algorithm was terminated after a maximum of MaxIter = 1000 iterations.

Cost Function: Every algorithm minimised the identical cost function CF defined in Eq. (5), which quantifies the maximum sidelobe level (SLL) of the CCAA.

Independent Runs: To account for stochastic variations, each algorithm was executed for 100 independent runs for every test case. The reported results (SLL, CPU time) are the mean values from these runs.

This standardised framework guarantees that any performance differences observed are attributable to the intrinsic search capabilities of the algorithms, not discrepancies in computational resources or problem formulation.

**Table 2:** Optimal control parameters for PSO and FPA algorithms.

PSO		FPA	
Population size	50	Population size	50
Inertia weight	0.7	Switching probability $\:p$	0.05–0.95
Cognitif acceleration coefficient	1.5	Uniform distribution $\:\epsilon\:$	[0–1]
Social acceleration coefficient	1.5	Scaling factor	0.1
Maximum velocity	2	Levy flight L($\:\beta\:$)	A
Maximum iteration	1000	Maximum iteration	1000

### Amplitude-only synthesis of CCAAs excluding the central element

**Fig. 2:**
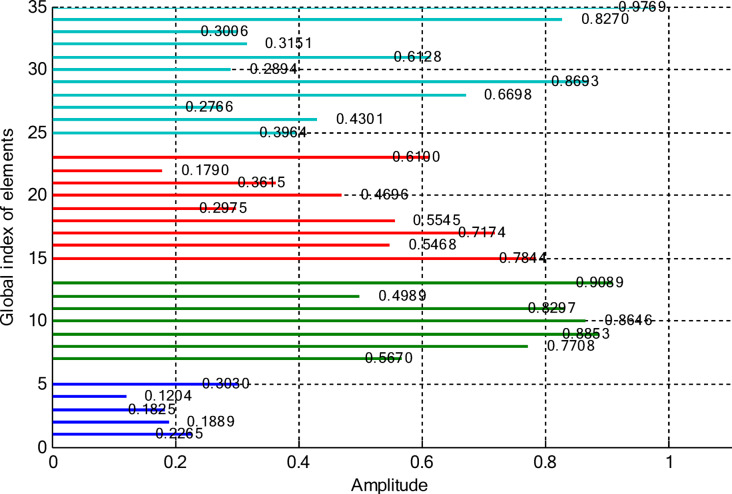
Amplitude distribution of CCAA with 5, 7, 9, and 11 elements optimized using the amplitude-only technique.

In this paper, two different non-uniform CCAA configurations with no central element are discussed. The first one contains 5, 7, 9, and 11 elements along the four rings, respectively, while the radial distances are 0.55λ, 0.75λ, 1.05λ, and 1.4λ. The other configuration has 8, 10, 12, and 14 elements along the radial distances 0.6λ, 0.8λ, 1.3λ, and 1.7λ. The excitation amplitudes in the range of 0.05 to 1.0 have been optimized for both designs using PSO, FPA, and the proposed optimization technique. All the excitation phases have been kept constant at 0° for amplitude-only synthesis. Figure 2 presents the amplitude distribution over the antenna elements for both the configurations. It is observed in both that the excitation of elements 1–5 falls in a range between 0.1 and 0.3 and can be considered under an inner ring, which does not greatly affect the main beam formation. The excitation of elements 6–13 remains between 0.5 and 0.9, which indicates that these elements are very vital for radiation pattern formation. Elements 14 to 22 show medium variation in their amplitude (0.17 to 0.78) and may be optimized for the sidelobe reduction. A huge variation in the amplitude spectrum (around 0.27 to 0.97) is obtained for the outer ring elements 23–32, indicating that these are also actively involved in improving the overall directivity.

**Fig. 3:**
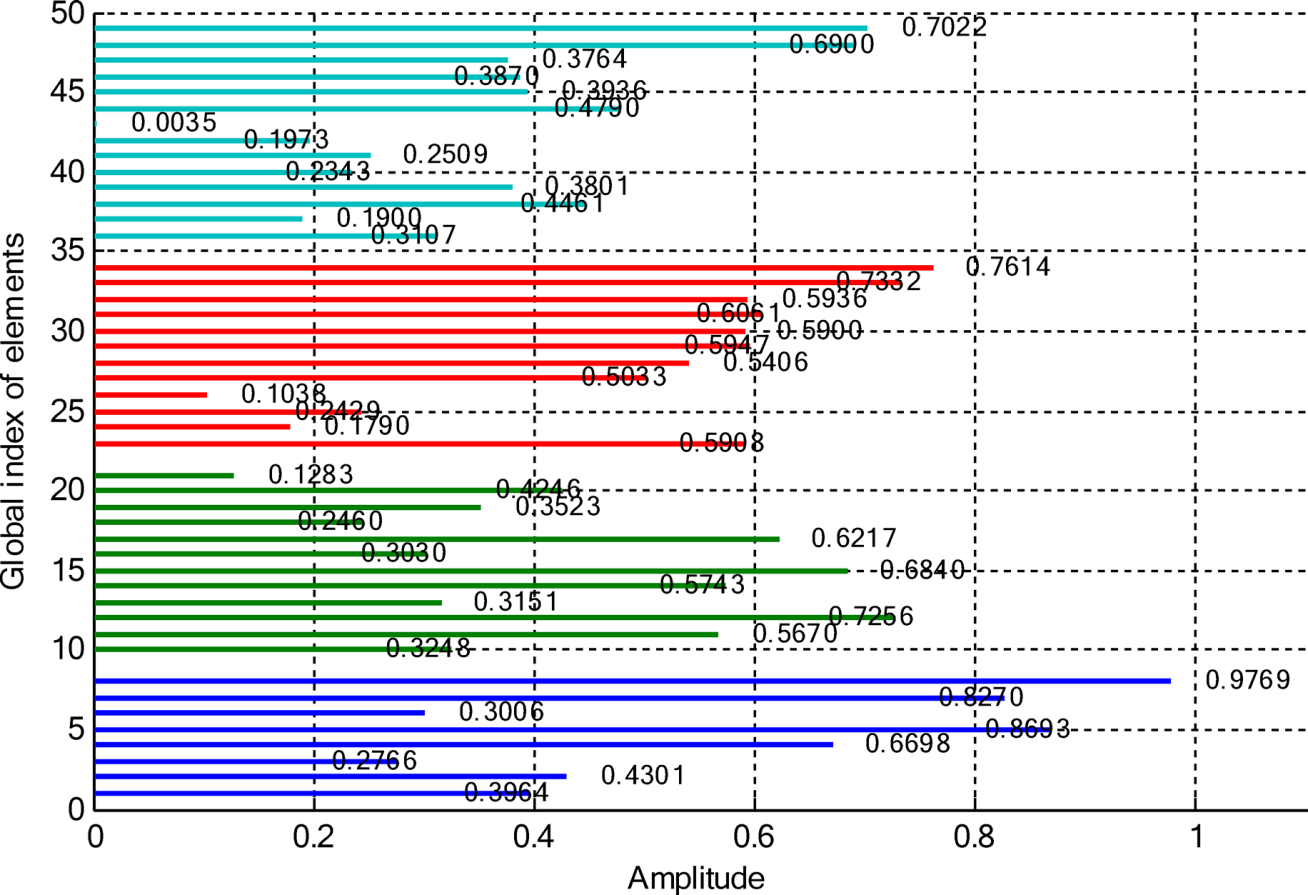
Optimized amplitude distribution for CCAA configurations with 8, 10, 12, and 14 elements using the amplitude-only approach.

**Table 3:** Array configuration, radius, SLL, and execution time for optimized CCAA using the proposed algorithm (Case I, excluding central element).

Configuration	Ring	Radius	SLL (dB)	Execution time (Sec)
	5	0.55	−40.48	9.47
	7	0.75		
	9	1.05		
	11	1.4		
	8	0.6	−41.17	13.19
	10	0.85		
	12	1.15		
	14	1.5		

**Fig. 4:**
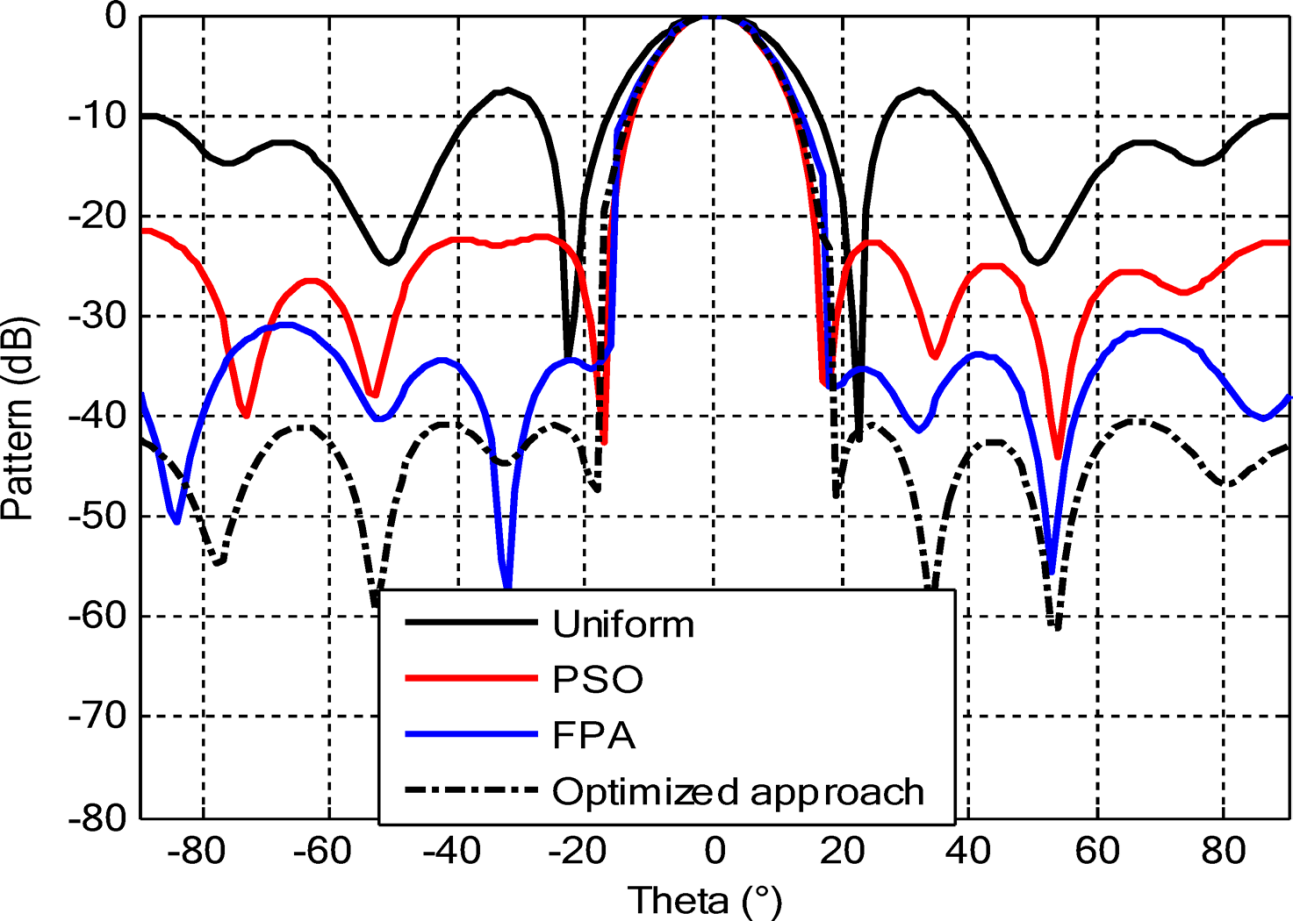
Beam patterns of optimized CCAA configurations with 5, 7, 9, and 11 elements in Set N° 1 using the amplitude-only technique via different algorithms (with no central element).

As seen from Fig. 3, the central rings (rings 1 and 2) are excited more and yield higher directivity values. On the other hand, the outer rings (rings 3 and 4) are excited at lower values, which contribute to directional pointing and interference reduction, hence offering lower sidelobe levels. Table 3 summarizes the results obtained by optimizing two CCAA configurations without a central element in terms of SLL and computational performance. In both designs, elements are symmetrically placed along the rings, and the number of elements increases with radial distance in order to increase the angular resolution. The corresponding radiation patterns of the two configurations are depicted in Figs. 4 and 6. The first configuration (5–7-9–11 elements at increasing radial distances) was optimized by using the proposed algorithm and also compared with PSO, FPA, and the uniform excitation technique. As illustrated in Fig. 4, the proposed approach achieved an extremely low SLL of − 40.48 dB, outperforming PSO (− 22.64 dB), FPA (− 31.49 dB), and the uniform excitation method (− 7.69 dB) for a directivity of 8.66 dB.

**Fig. 5:**
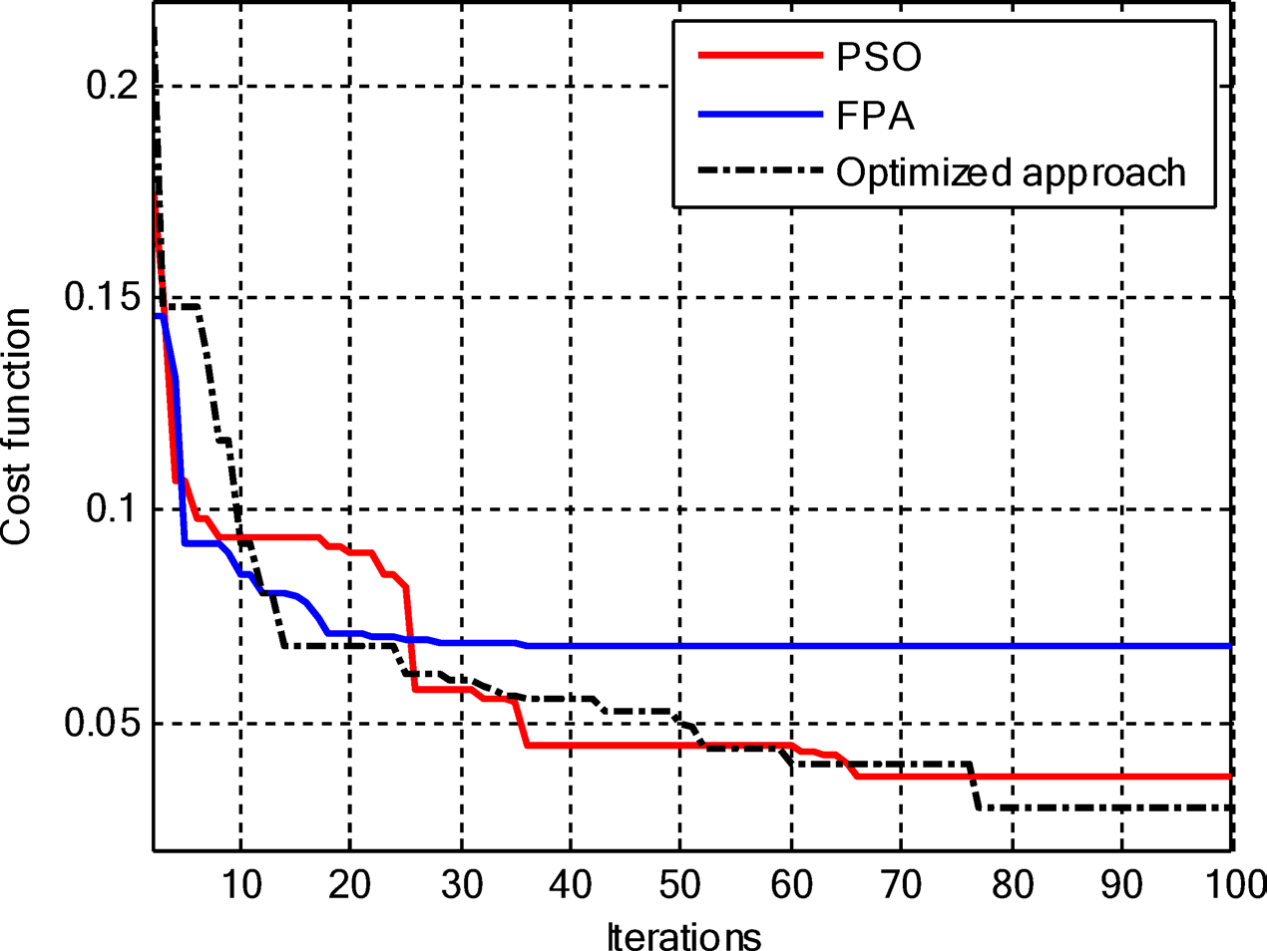
Convergence curve of CCAA for Case I (Set I).

Figure 5 compares the convergence behaviours for the three techniques. It indicates that the proposed hybrid PSO–FPA consistently achieves the lowest cost value and thus the best radiation performance. PSO provides gradual improvement but converges more slowly and to a higher final cost, while FPA converges quickly but becomes trapped on a higher plateau. Overall, the hybrid method combines the fast initial descent of FPA with the refined search of PSO, yielding faster and more effective convergence. In the second configuration, the proposed hybrid PSO–FPA maintains a consistently lower cost value than standard PSO and FPA over the entire iteration range, confirming its better optimisation capability for amplitude-only CCAA synthesis without the central element. The hybrid curve exhibits a rapid early descent followed by steady refinement, whereas PSO converges more slowly and FPA stagnates at a higher plateau, indicating that the hybrid strategy achieves superior sidelobe suppression and pattern quality with improved convergence behaviour as shown in Fig. 7.

**Fig. 6:**
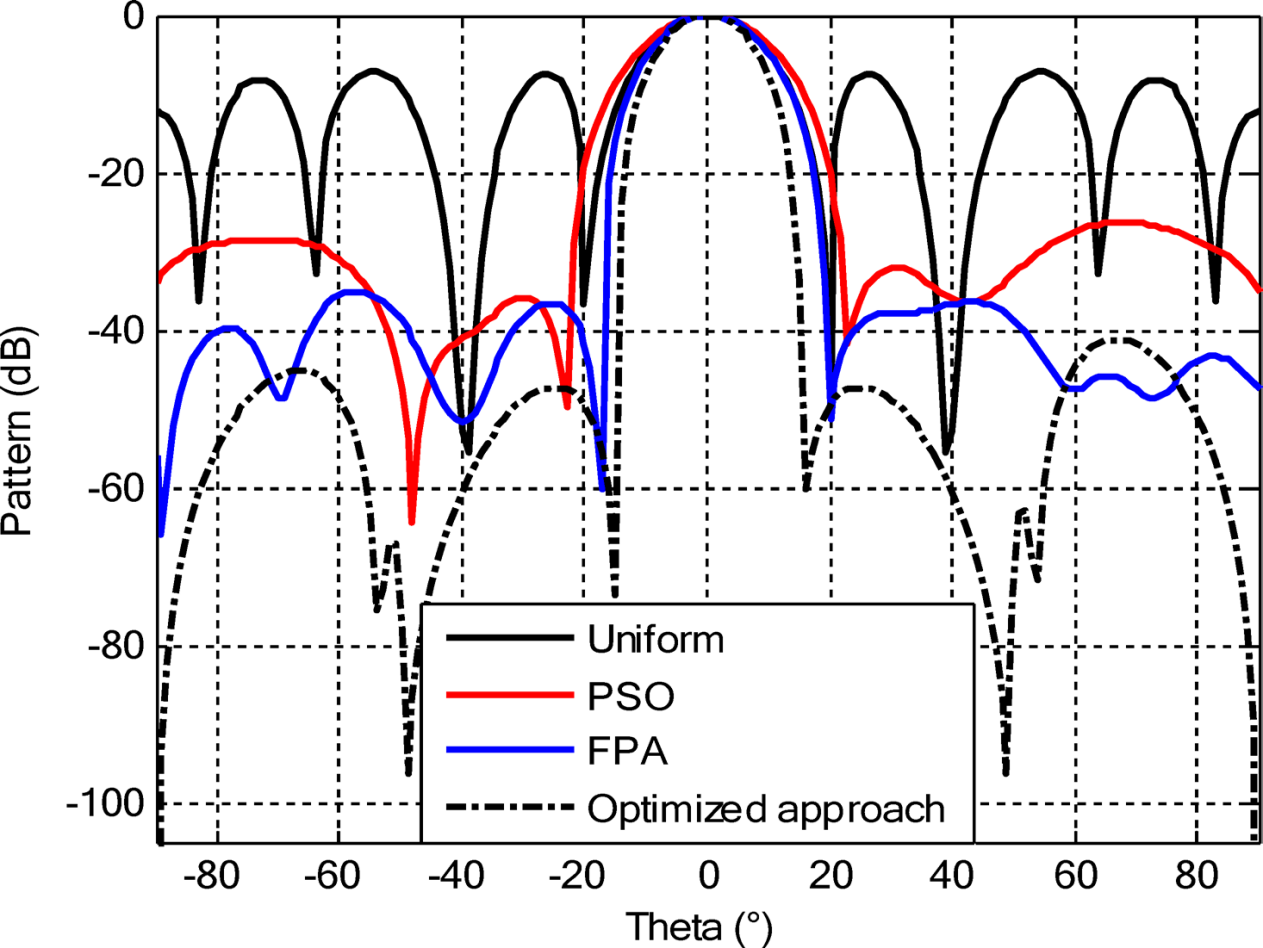
Beam patterns for Set N° 2 CCAA configurations with 8, 10, 12, and 14 elements optimized using the amplitude-only technique by various algorithms (without central element).

**Fig. 7:**
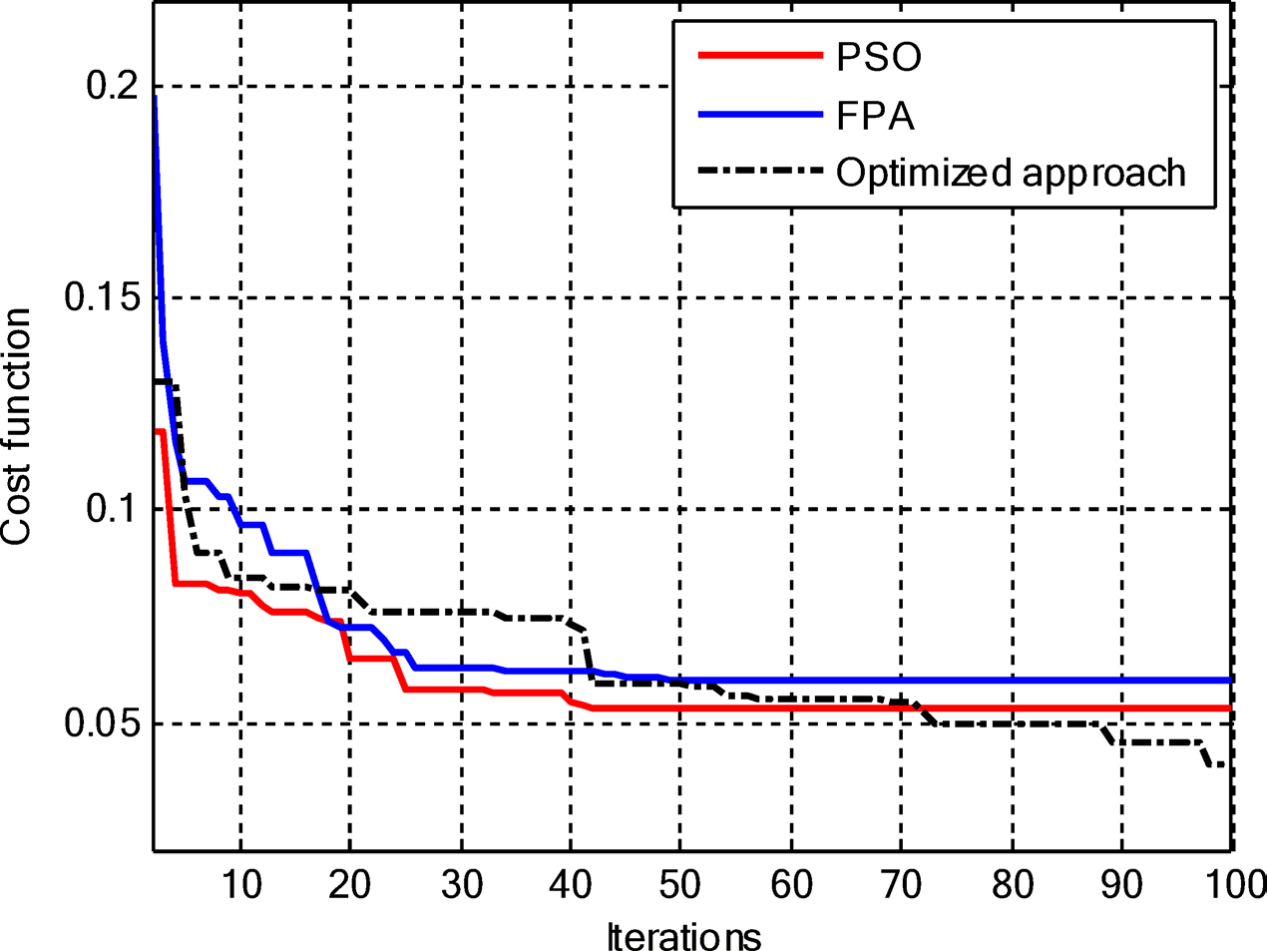
Convergence curve of CCAA for Case I (Set II).

In the second configuration, the array consists of 8, 10, 12, and 14 elements in each ring, which are positioned more closely at radii of 0.6λ, 0.85λ, 1.15λ, and 1.5λ, respectively. This dense-element distribution generates a better directivity of 8.87 dB owing to a more coherent phase front, while introducing stronger side lobe levels. Even under these circumstances, the proposed algorithm synthesizes a superior side lobe suppression with SLLs of − 41.17 dB compared to FPA (− 35.11 dB), PSO (− 26.36 dB), and uniform excitation (− 7.16 dB), as depicted in Fig. 7. Table 4 illustrates the superiority of the proposed hybrid algorithm in the synthesis of CCAA configurations in Case I. It produces much lower maximum SLLs, − 40.48 dB for Set I and − 41.17 dB for Set II, unlike Artificial Bee Colony (ABC)^42^ and Whale Optimization Algorithm (WOA)^4^, with values that lie within the range of − 27 dB to − 32 dB.

The proposed hybrid PSO-FPA algorithm is assessed against four recognised nature-inspired metaheuristics to guarantee a thorough and equitable comparison. The choice of these benchmarks is intentional, commencing with the standard PSO and FPA as the fundamental elements of the hybrid. Direct comparison with them substantiates the synergy of the hybridisation and quantifies the enhancement over the independent algorithms. The Artificial Bee Colony (ABC) algorithm is incorporated to contest the method with various competitive search strategies due to its strong exploration capabilities derived from honeybee foraging, while the Whale Optimisation Algorithm (WOA) is utilised as a modern benchmark recognised for its efficient equilibrium between exploration and exploitation, emulating humpback whale behaviour. This collection serves as a comprehensive evaluation of traditional and contemporary algorithms with various search strategies, convincingly illustrating the superiority and resilience of the suggested PSO-FPA approach. These results confirm the effectiveness of the proposed method for enhancing sidelobe suppression and overall array performance.

**Table 4:** Comparison of maximum SLL (in dB) for optimized CCAA configurations in case I.

Set No.	Optimization method	Maximum SLL (dB)
I	ABC^40^	−29
	WOA^41^	−27
	Proposed method	−40.48
II	ABC^40^	−32
	WOA^41^	−31
	Proposed method	−41.17

Table 5 demonstrates that the proposed hybrid PSO-FPA algorithm achieves a dominant Pareto front by consistently synthesising concentric circular arrays with ultra-low sidelobes (below − 40 dB) in substantially less CPU time (25–36% faster) than standard PSO and FPA. This dual improvement in both final performance and convergence speed validates the synergy of the hybrid approach and underscores its practical value for rapid, high-performance antenna design.

**Table 5:** CPU comparison of algorithms vs. SLL metrics for case I.

Case No.	Set No.	Algorithm	CPU Time (s)	SLL (dB)
I	I	PSO	12.62	−22.78
		FPA	14.94	−30.97
		Proposed method	9.47	−40.48
	II	PSO	15.67	−26.36
		FPA	16.09	−35.09
		Proposed method	13.9	−41.17

### Amplitude-only synthesis of CCAAs including the central element

**Fig. 8:**
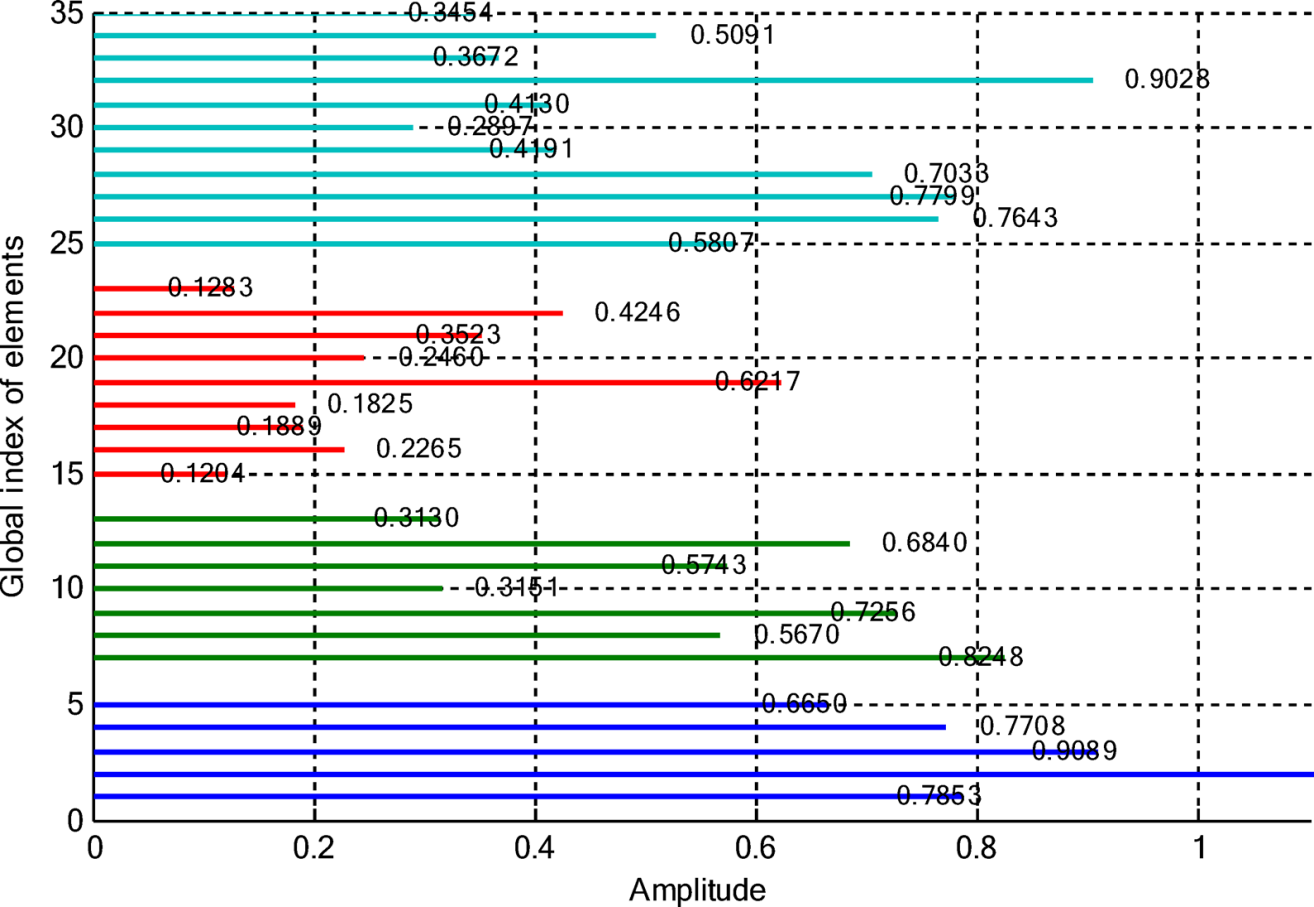
Amplitude distribution of CCAA with 5, 7, 9, and 11 elements using the amplitude-only technique with a central element.

**Fig. 9:**
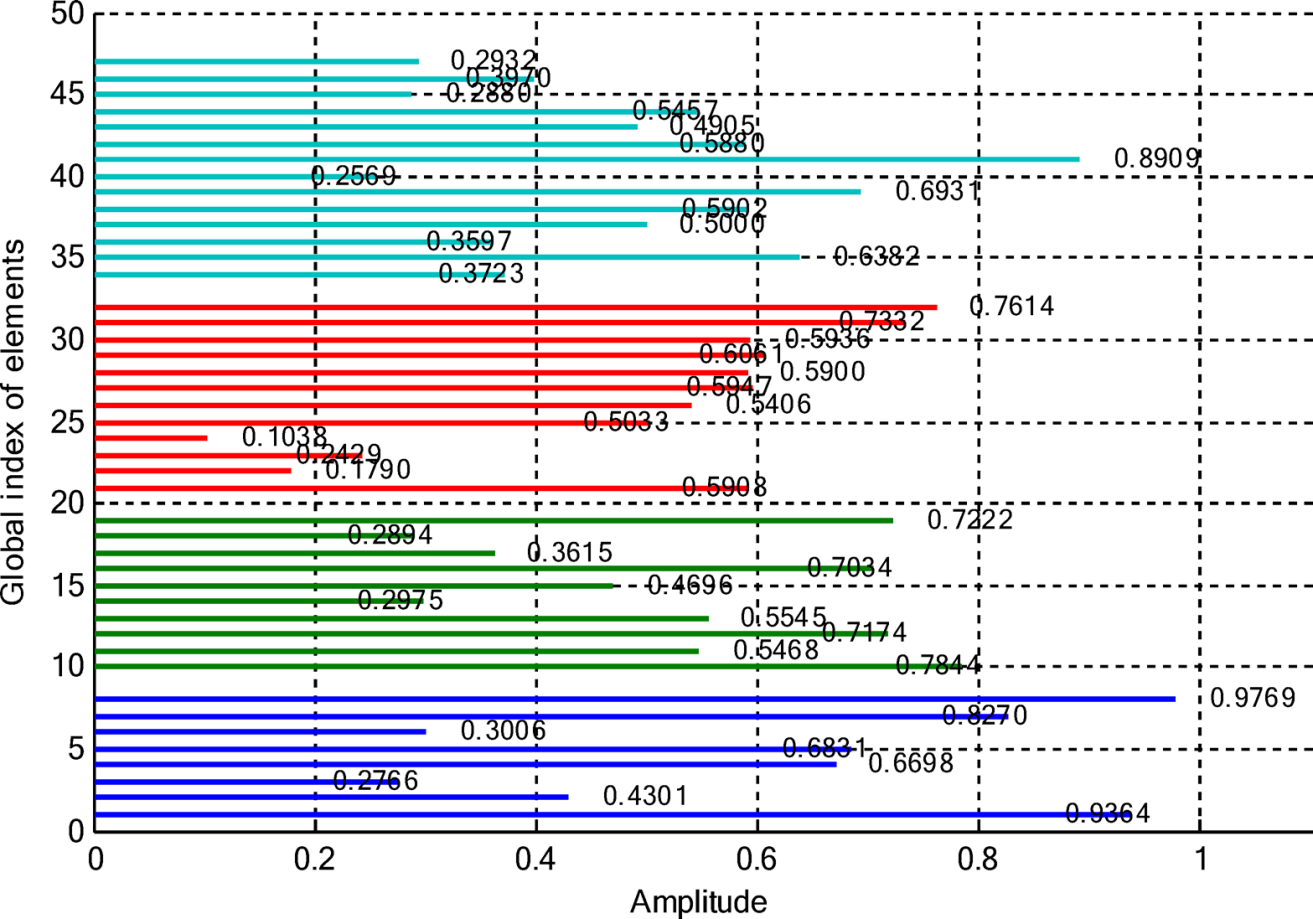
Detailed amplitude distribution in CCAA arrays with 8, 10, 12, and 14 elements, implementing the amplitude-only technique with a central component for optimized performance.

Figures 8 and 9 present the amplitude excitation profiles for two distinct CCAA configurations, each comprising four rings. The first configuration features rings populated with 5, 7, 9, and 11 elements, located at radial distances of 0.55λ, 0.75λ, 1.05λ, and 1.4λ, respectively. The second array includes 8, 10, 12, and 14 elements placed at 0.6λ, 0.8λ, 1.3λ, and 1.7λ. Notably, the innermost ring elements (indices 1–6 at ~ 0.55λ) exhibit low excitation amplitudes ranging from 0.1 to 0.3, indicating minimal impact on directional control. As we progress radially outward, elements 7 to 14, occupying intermediate positions between 0.75λ and 1.05λ, demonstrate heightened excitation levels (0.5–0.9), underscoring their critical role in main-lobe beam shaping. Beyond this, elements on the outer rings (15–24 at 1.3λ–1.7λ) exhibit a broader excitation range (0.17–0.78), likely tuned for effective sidelobe attenuation.

**Fig. 10:**
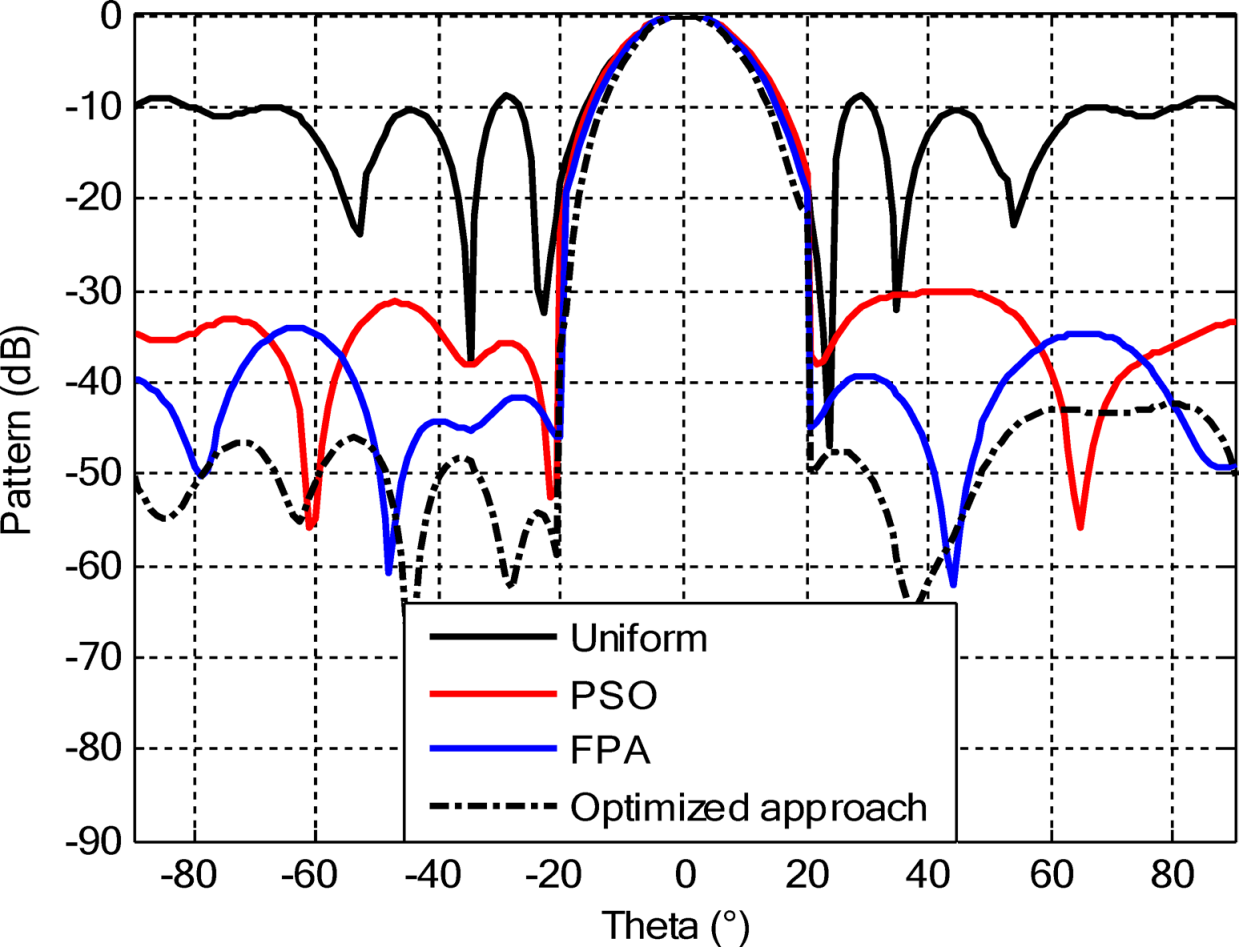
Beam patterns for Set N°1 (5, 7, 9, 11) optimized CCAA using the amplitude-only technique, achieved by various algorithms with a central element.

The outermost elements (indices 25–36) exhibit a dispersed amplitude distribution (~ 0.27 to ~ 0.97), implying that their excitation has been precisely optimized to reinforce directivity and suppress sidelobes in off-boresight directions. Table 6 presents the optimal excitation weights and some relevant performance figures obtained by the proposed amplitude-only synthesis algorithm in Case II for Sets I and II. The synthesized configurations attained good trade-offs among excitation uniformity, element spacing, SLL, and computational efficiency. Relevant far-field radiation patterns are provided in Figs. 10 and 12. It is seen that the presence of a central element remarkably affects the radiation behavior of the arrays. The proposed approach results in a main beam directivity of 11.70 dB in Set I and 13.14 dB in Set II. However, such an increased directivity is attained at the price of higher SLL, which reaches − 42.56 dB in one of the synthesized configurations.

**Table 6:** Optimized CCAA configuration, radius, SLL, and execution time for case II (with central element) using the suggested algorithm.

Configuration	Ring	Radius	SLL (dB)	Execution time (Sec)
	5	0.55	−42.56	11.08
	7	0.75		
	9	1.05		
	11	1.4		
	8	0.6	−44.08	14.09
	10	0.85		
	12	1.15		
	14	1.5		

The comparative analysis reveals that although the conventional metaheuristic approaches, namely the FPA and PSO, achieve reduced SLLs of −34.62 dB and − 30.14 dB, respectively, (Fig. 8) with a compromise in directivity, in the second case (Fig. 9) the proposed algorithm achieves a more pronounced SLL suppression at −44.08 dB, outperforming both FPA (−30.84 dB) and PSO (−26.53 dB). The comparison underlines the superiority of the suggested approach in minimizing sidelobes without compromising beam focus. Nevertheless, it should be mentioned that including a central element may introduce mutual coupling effects, which can definitely alter the excitation distribution and radiation pattern. If such coupling is not properly managed, it would assuredly have an adverse impact on sidelobe performance, an effect which can be observed in pattern distortions, highlighted in Fig. 10.

**Fig. 11:**
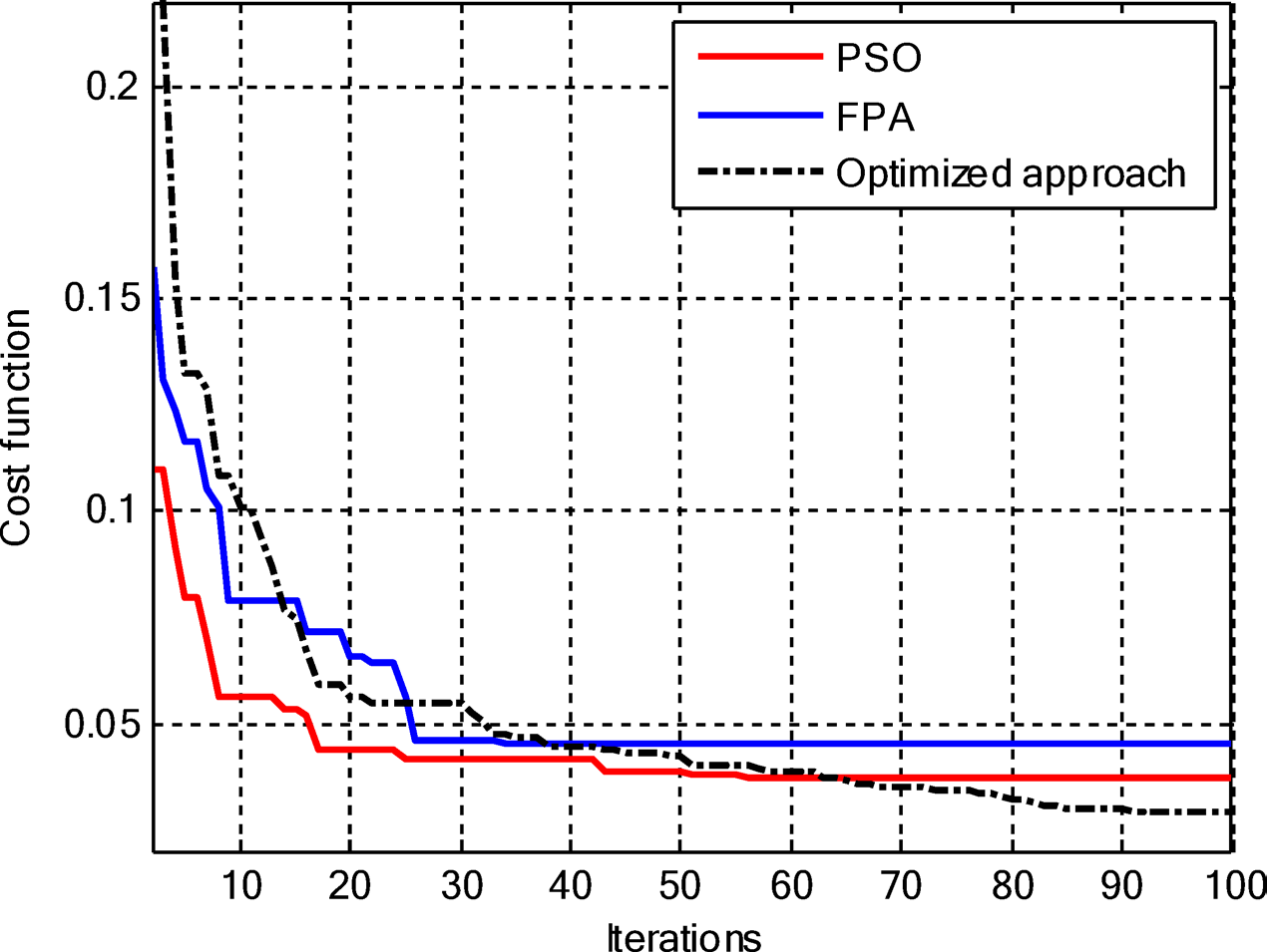
Convergence curve of CCAA for Case II (Set I).

Including the central element, the proposed hybrid PSO–FPA still converges to the lowest cost among the three methods as shown in Fig. 11, but now its superiority becomes more evident in the later iterations as it continues improving while PSO and FPA saturate. In the early stages, PSO and FPA reduce the cost more quickly, yet they stall at higher cost levels, whereas the hybrid algorithm gradually exploits the additional degree of freedom offered by the central element to further suppress sidelobes and refine the beam shape, resulting in the best overall radiation performance.

Table 7 compares the maximum SLL obtained using different optimization methods in Case II of the CCAA. Among these, for Set I, the ABC method^42^ was able to provide a maximum SLL of −32 dB, whereas the WOA in^4^ improved this to −30.5 dB. However, the proposed method turns in a much better performance with a maximum SLL of −42.56 dB, which ensures much better sidelobe suppression. For Set II, the ABC method performs at a maximum SLL of −28 dB, which is better than Set I but still higher than the value achieved with the proposed method. The WOA achieves − 31 dB for Set II, which further improves the performance obtained in Set I, yet remains higher than the value achieved by the proposed method. Again, the best performance is provided by the proposed technique, yielding a maximum SLL of −44.08 dB, proving its superiority in terms of sidelobe reduction and optimization. From the above, it can be concluded that the proposed method has outperformed both the ABC and WOA techniques in minimizing the sidelobes, and the performance is slightly enhanced for Set II compared to Set I.

**Fig. 12:**
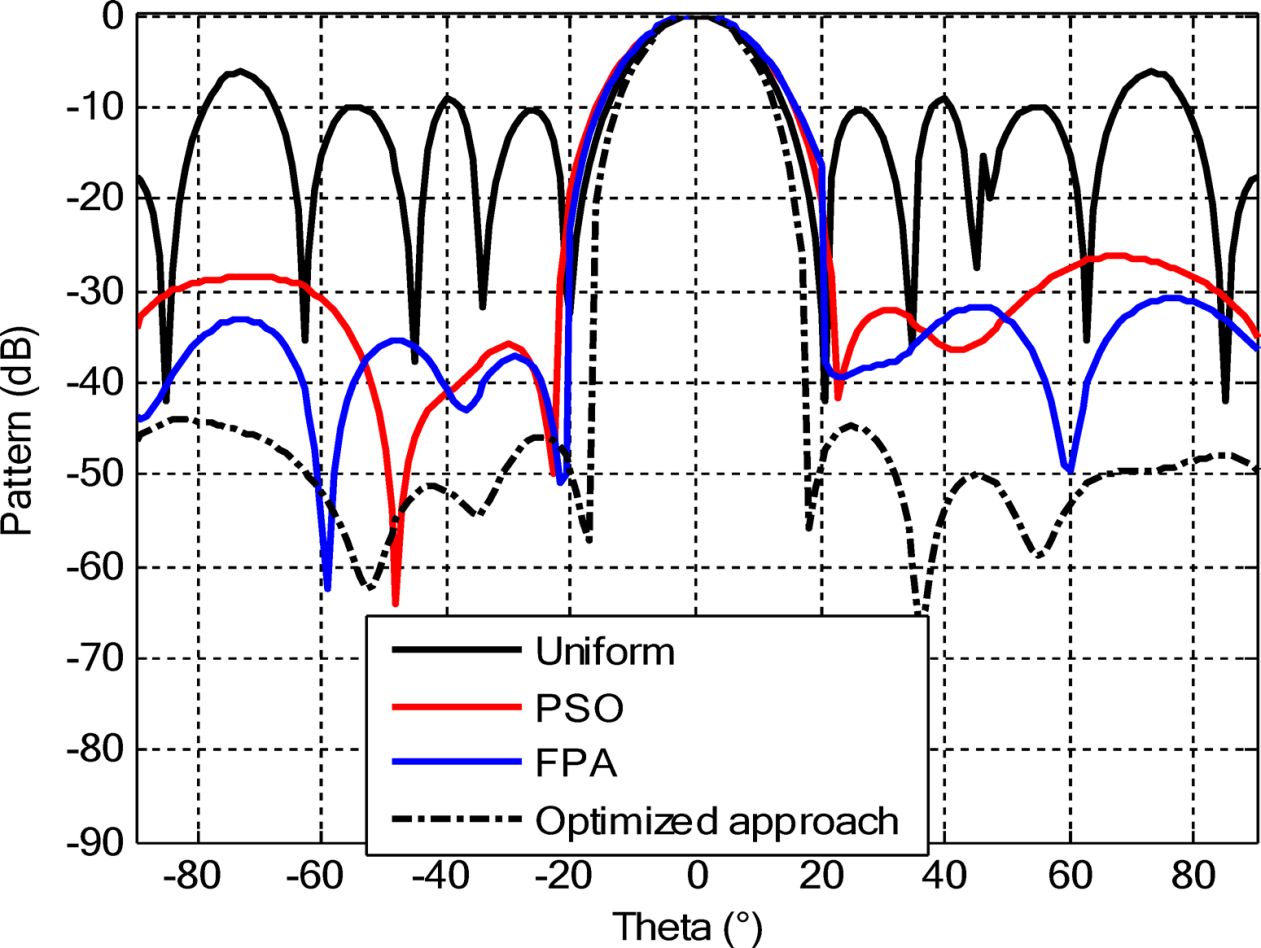
Beam patterns of optimized CCAA for Set N°2 (8, 10, 12, 14) using the amplitude-only technique with a central element across various optimization algorithms.

**Fig. 13:**
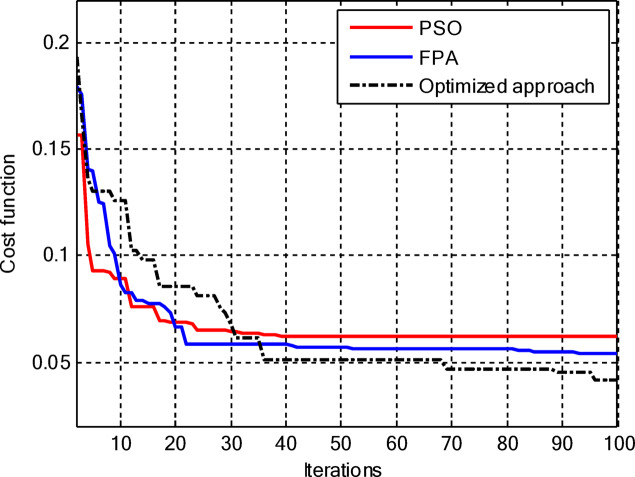
Convergence curve of CCAA for Case II (Set II).

Figure 13 shows that for amplitude-only synthesis of CCAAs, the hybrid PSO–FPA is more effective than PSO and FPA in achieving the lowest final cost. The FPA progresses steadily but insufficiently, while the PSO has a quick initial drop but stabilises too early. After about 40 cycles, the hybrid method combines local refinement and global exploration, demonstrating resilience when optimising CCAA structures.

**Table 7:** Comparison of maximum SLL (in dB) achieved by different optimization methods for CCAA in case II.

Set No.	Optimization method	Maximum SLL (dB)
I	ABC^30^	−32
	WOA^31^	−30.5
	Proposed method	−42.56
II	ABC^30^	−28
	WOA^31^	−31
	Proposed method	−44.08

Based on the comprehensive results from Case II presented in Table 8, the proposed hybrid PSO-FPA algorithm again demonstrates unequivocal superiority. It consistently achieves the deepest nulls in sidelobe levels (reaching an exceptional − 44.08 dB) while simultaneously maintaining the shortest computation times. This performance represents a critical dual advantage: it is approximately 20–34% faster than the benchmark algorithms while improving sidelobe suppression by a substantial 8 to 18 dB. This consistent trend across multiple test cases solidifies the algorithm’s robustness and validates its practical efficacy for the rapid synthesis of high-performance antenna arrays.

**Table 8:** CPU comparison of algorithms vs. SLL metrics for case II.

Case No.	Set No.	Algorithm	CPU Time (s)	SLL (dB)
II	I	PSO	14.78	−30.16
		FPA	16.83	−34.07
		Proposed method	11.08	−42.56
	II	PSO	15.01	−26.43
		FPA	17.52	−30.84
		Proposed method	14.09	−44.08

### Amplitude and ring radius-based synthesis of CCAAs without a central element

**Fig. 14:**
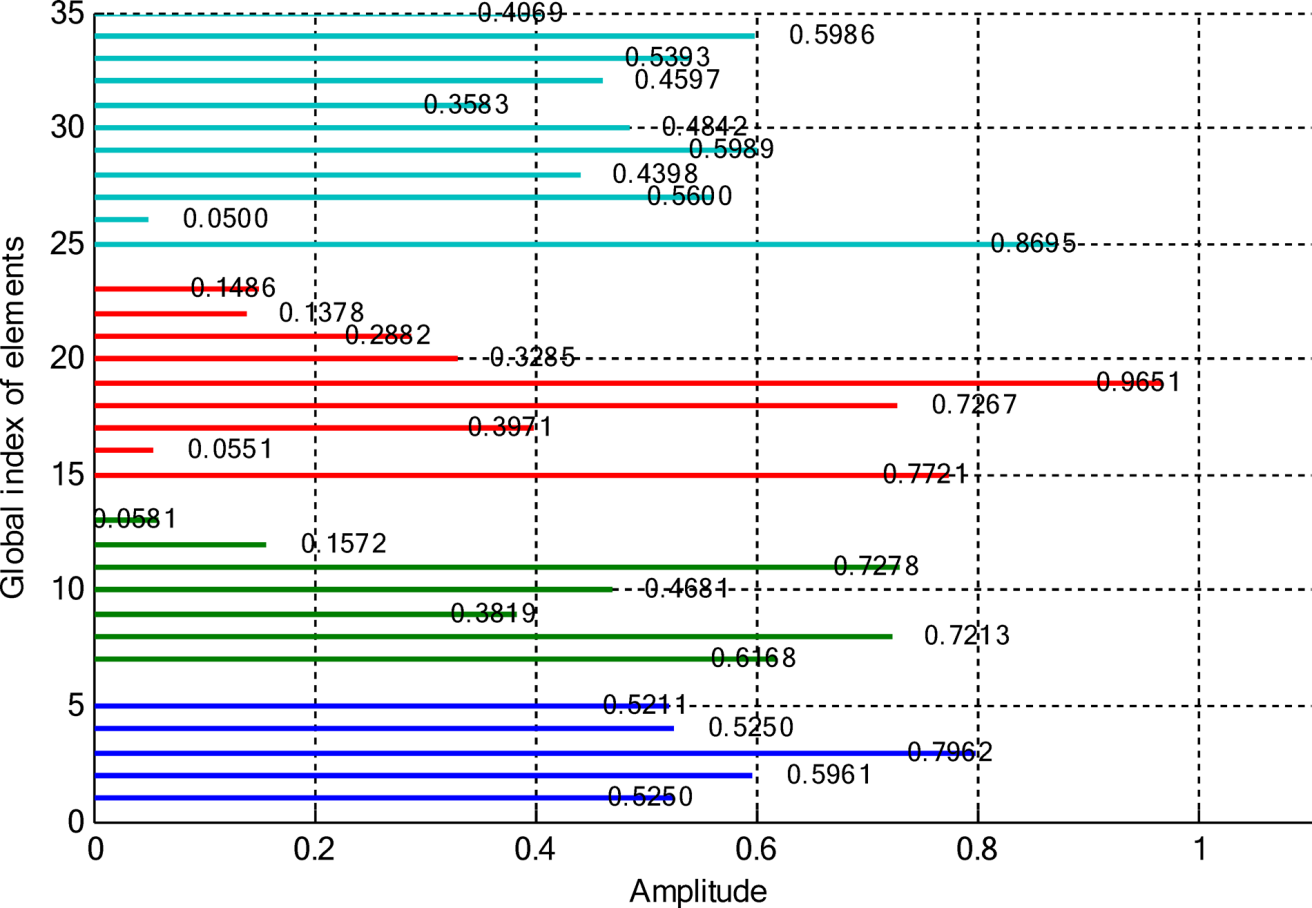
Optimized amplitude distribution of a CCAA with 5, 7, 9, and 11 elements per ring using joint amplitude and ring radius optimization.

**Fig. 15:**
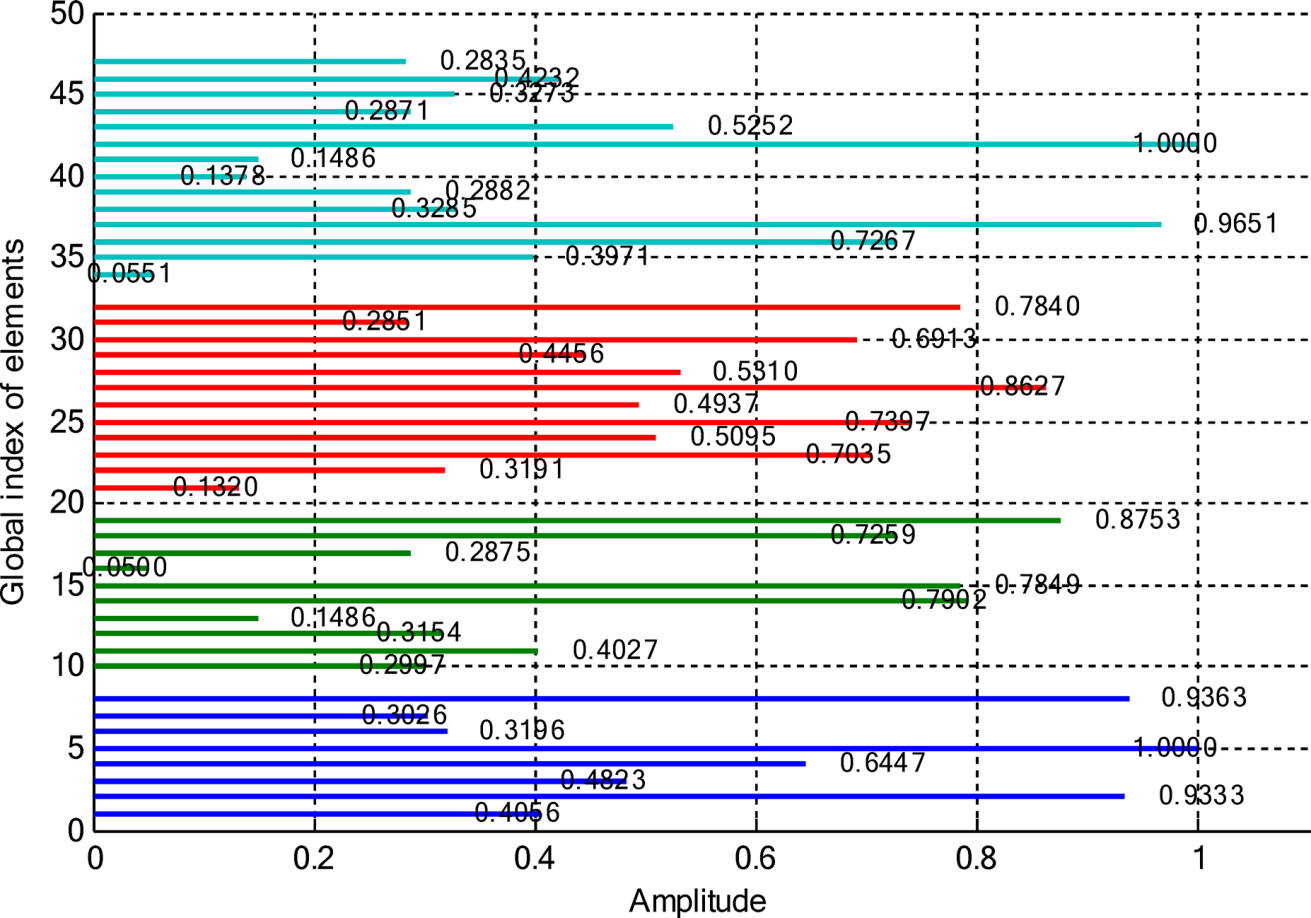
Amplitude profiles of a ring radius and excitation-optimized CCAA with 8, 10, 12, and 14 elements per ring.

This section deals with the optimization-based synthesis approach for CCAAs without any central element. Particular interest has gone into the joint optimization of excitation amplitudes as well as ring radii for improved radiation performance. Optimized amplitude distributions for two CCAA configurations obtained by using a hybrid technique that simultaneously optimizes amplitude weights and ring radii are shown in Figs. 14 and 15. Figure 14 refers to a configuration with 5, 7, 9, and 11 elements per ring (for a total of 32 elements), whereas Fig. 11 refers to a denser configuration with 8, 10, 12, and 14 elements per ring (for a total of 44 elements). In Fig. 14, the excitation amplitudes vary from as low as 0.05 (for example, elements 13 and 22) up to 0.97 for element 23. This points out a strong tapering strategy in order to reduce sidelobes. The inner ring elements, such as elements 2 and 5, have moderate amplitudes (≈ 0.52–0.60), whereas some elements of the outermost ring (for example, 23 and 27) are highly excited to provide more directivity.

In contrast, Fig. 15 shows a much more involved and asymmetric amplitude profile, which varies between 0.05 for elements 15 and 31, and 1.00 for elements 5 and 42. Such variation indicates that the optimizer has taken advantage of increased spatial resolution. Significantly, the amplitudes of mid-ring elements, such as 18 and 20, are about 0.87 and 0.70, respectively, while outer elements like 42 and 43 are fully excited to enhance main-lobe strength. A wider range of excitation in this more compact configuration underscores the optimizer’s ability to fine-tune array behavior for enhanced sidelobe control and beam directivity. Table 9 lists the optimized ring radii for two CCAA configurations without a central element. Optimized radii for the first configuration with 5, 7, 9, and 11 elements in each ring are 0.8588, 1.2000, 1.5696, and 1.9637, respectively. For the second configuration with 8, 10, 12, and 14 elements in each ring, the optimized radii are 0.9055, 1.1705, 1.4305, and 1.4695, respectively. This has been achieved with an effective optimization strategy aimed at maximizing directivity while suppressing SLLs effectively without having a central element.

**Table 9:** Optimized CCAA configurations for case III (without central element): element distribution, ring radii, SLL, and execution time using the proposed algorithm.

Configuration	Ring	Radius	SLL (dB)	Execution time (Sec)
	5	0.8588	−45.01	11.30
	7	1.2000		
	9	1.5696		
	11	1.9637		
	8	0.9055	−44.88	11.95
	10	1.1705		
	12	1.4305		
	14	1.4695		

**Fig. 16:**
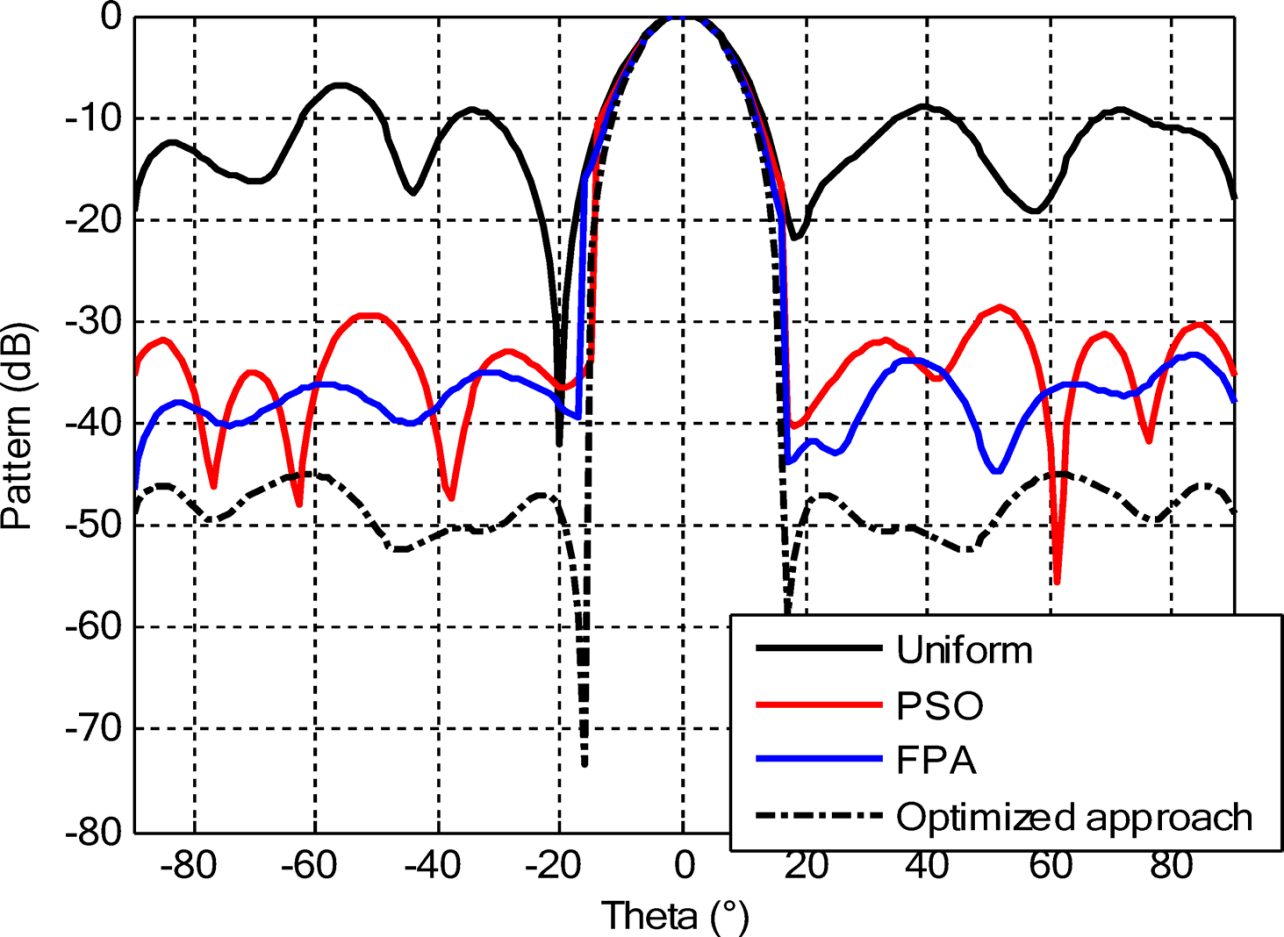
Beam patterns for Set N°1 (5, 7, 9, 11 elements per ring) CCAA optimized via joint amplitude and ring radius tuning using various algorithms (excluding central element).

Figures 16 and 18 show the synthesized beam patterns for the two CCAA configurations when using four different excitation strategies, namely uniform excitation, PSO, FPA, and the hybrid optimization technique proposed in this letter. For Set N°1 (5, 7, 9, and 11 elements per ring, Fig. 16), the most effective performance is obtained by the hybrid method, which reaches sidelobe levels below − 40 dB. Moderate improvements in beam shaping are achieved by PSO and FPA, though they present limitations in the sidelobe suppression and main beam sharpness. As expected, the poorest results are obtained with uniform excitation, characterized by broad main lobes and significant sidelobe levels. In Fig. 18, corresponding to Set N°2 (8, 10, 12, and 14 elements per ring), the superiority of the hybrid optimization technique is still more emphasized. It reaches sidelobe suppression below − 45 dB and gives a narrower, highly symmetric main beam, features that are very desirable for any application where high directivity is requested.

**Fig. 17:**
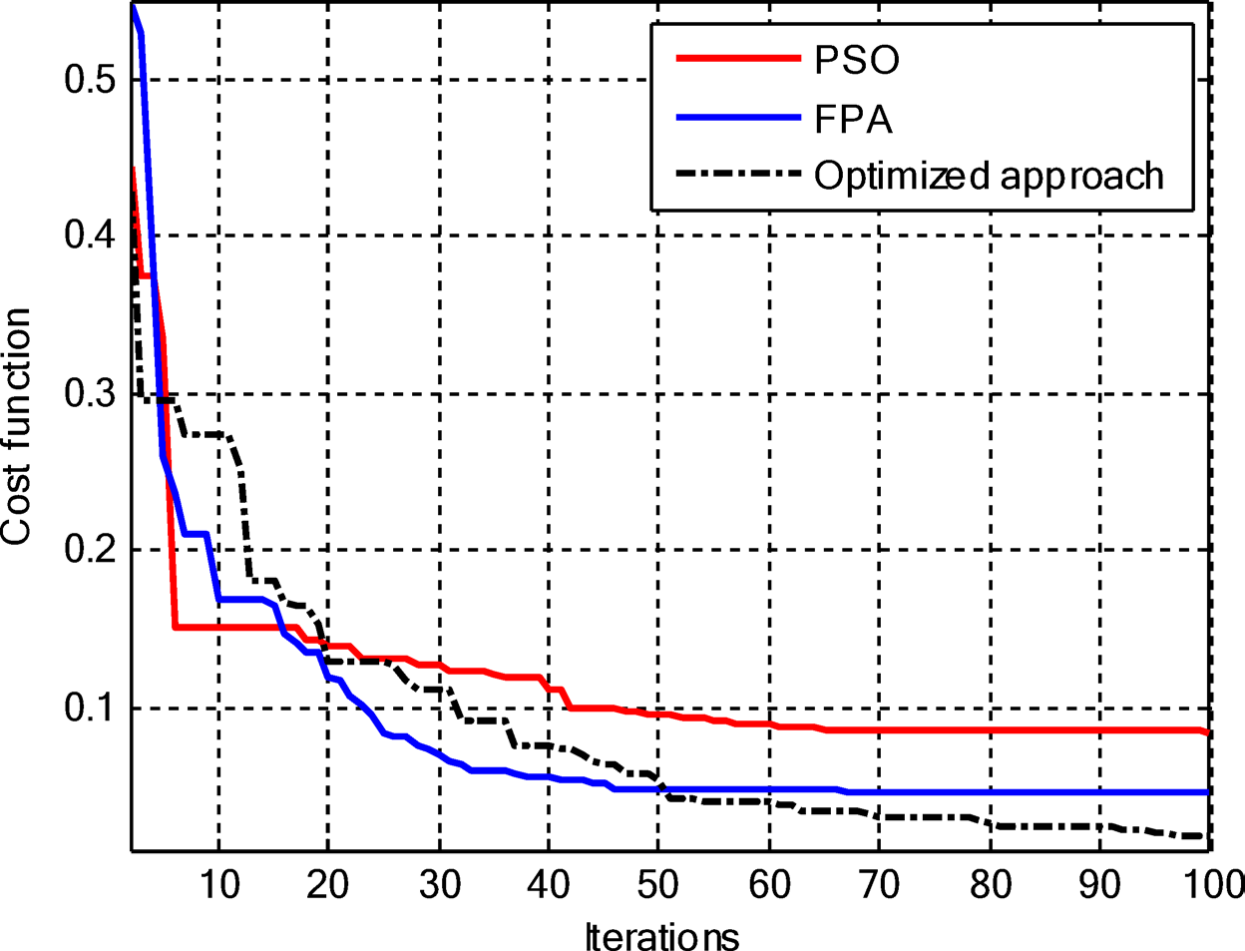
Convergence curve of CCAA for Case III (Set I).

**Fig. 18:**
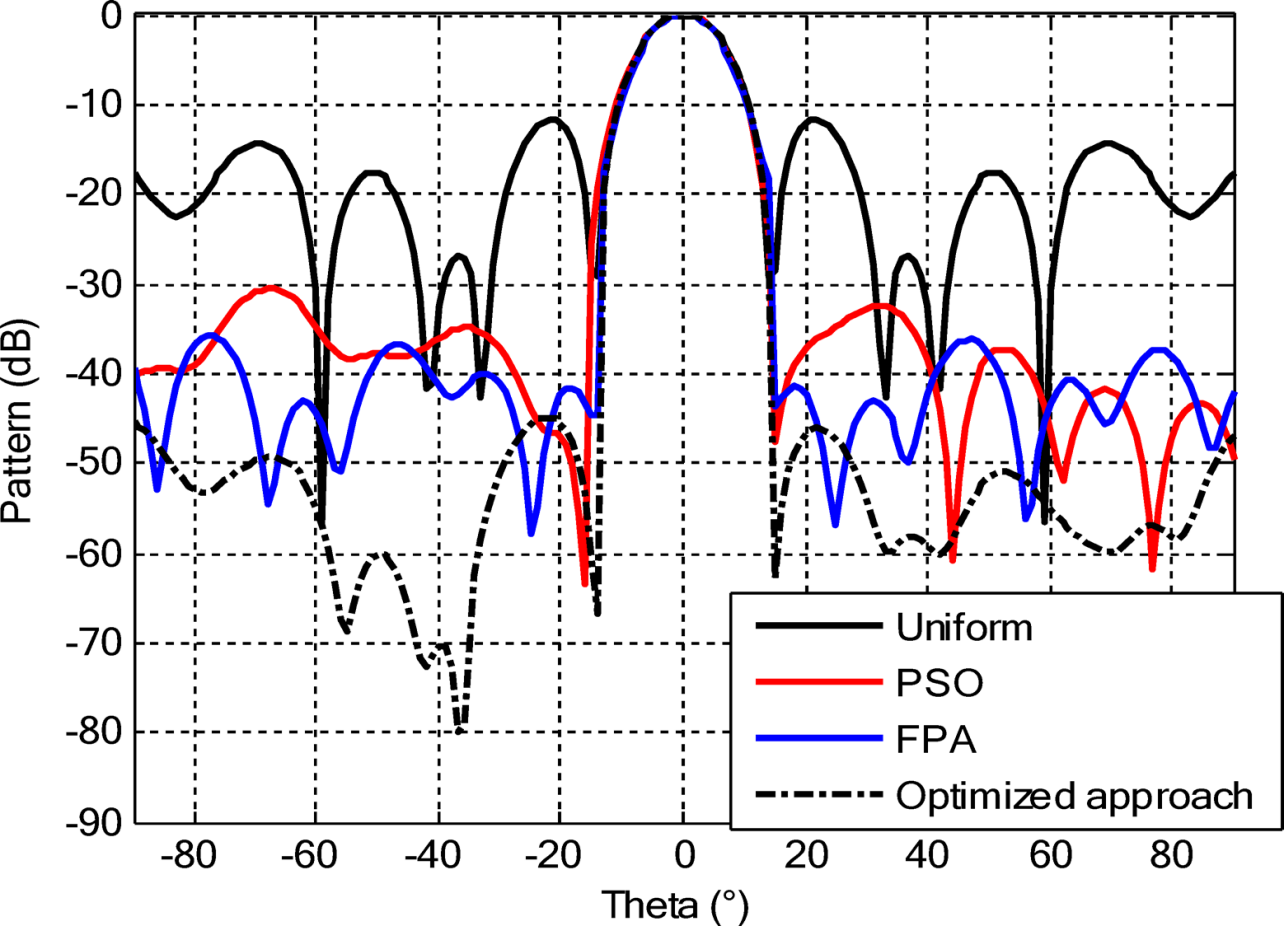
Optimized beam patterns for Set N°2 (8, 10, 12, 14 elements per ring) in CCAA using combined amplitude and ring radius optimization via multiple algorithms (no central element).

Figure 17 depicts the convergence for the synthesis of joint amplitude and ring radius of CCAAs devoid of a central element, wherein the hybrid PSO–FPA achieves the minimal final cost, surpassing both PSO and FPA. Particle Swarm Optimisation (PSO) demonstrates a swift beginning decline but stabilises around the 20th iteration at a suboptimal value of approximately 0.15, while the Firefly Algorithm (FPA) displays a more gradual decrease with greater stagnation at around 0.10; the hybrid approach amalgamates these advantages, achieving continual enhancement below 0.05 by the 100th iteration. This diagram illustrates improved optimisation potential when simultaneously adjusting geometric and excitation parameters in the more intricate no-centre design

**Fig. 19:**
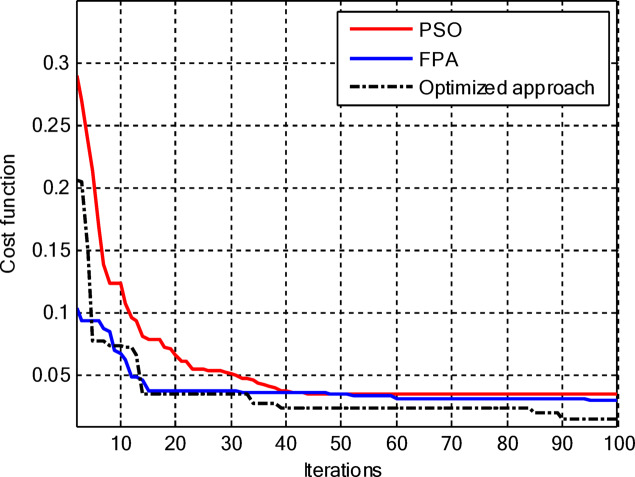
Convergence curve of CCAA for Case III (Set II).

Figure 19 illustrates that, for the synthesis of amplitude and ring radius in CCAAs with a central element for Set II, the hybrid PSO–FPA achieves the lowest final cost (~ 0.05), outperforming PSO (~ 0.10 level after iteration 20) and FPA (~ 0.08, a state of stagnation). PSO experiences a significant initial decline but stabilises early, whereas FPA advances consistently but not optimally; the hybrid method utilises joint geometric excitation optimisation through a combined exploration-exploitation approach to achieve enhanced convergence in this complex configuration.

As element density increases, the difference in performance between the hybrid approach and other methods investigated-uniform, PSO, and FPA-becomes greater, and hence the scalability and robustness of simultaneous optimization of both excitation amplitudes and ring radii are highlighted. In general, these results demonstrate that even for structures without a central element, hybrid optimization techniques can substantially improve CCAA performance by providing accurate beam control together with lower sidelobe levels and enhanced directivity.

The results for Case III (Table 10) confirm the definitive and consistent trend established across all experiments. The proposed hybrid PSO-FPA algorithm achieves a remarkable sidelobe level near − 45 dB, representing an improvement of approximately 10–15 dB over the benchmark algorithms. This is accomplished with a comparable or slightly lower CPU time, demonstrating a superior convergence profile. The repeated evidence across three distinct cases solidifies the algorithm’s robustness and reliability, making it a compelling and efficient solver for synthesizing ultra-low sidelobe concentric circular arrays.

**Table 10:** CPU comparison of algorithms vs. SLL metrics for case III.

Case No.	Set No.	Algorithm	CPU Time (s)	SLL (dB)
III	I	PSO	12.08	−28.71
		FPA	13.99	−33.87
		Proposed method	11.30	−45.01
	II	PSO	13.88	−30.58
		FPA	14.64	−35.84
		Proposed method	11,95	−44.88

### Amplitude and ring radius-based synthesis of CCAAs with a central element

**Fig. 20:**
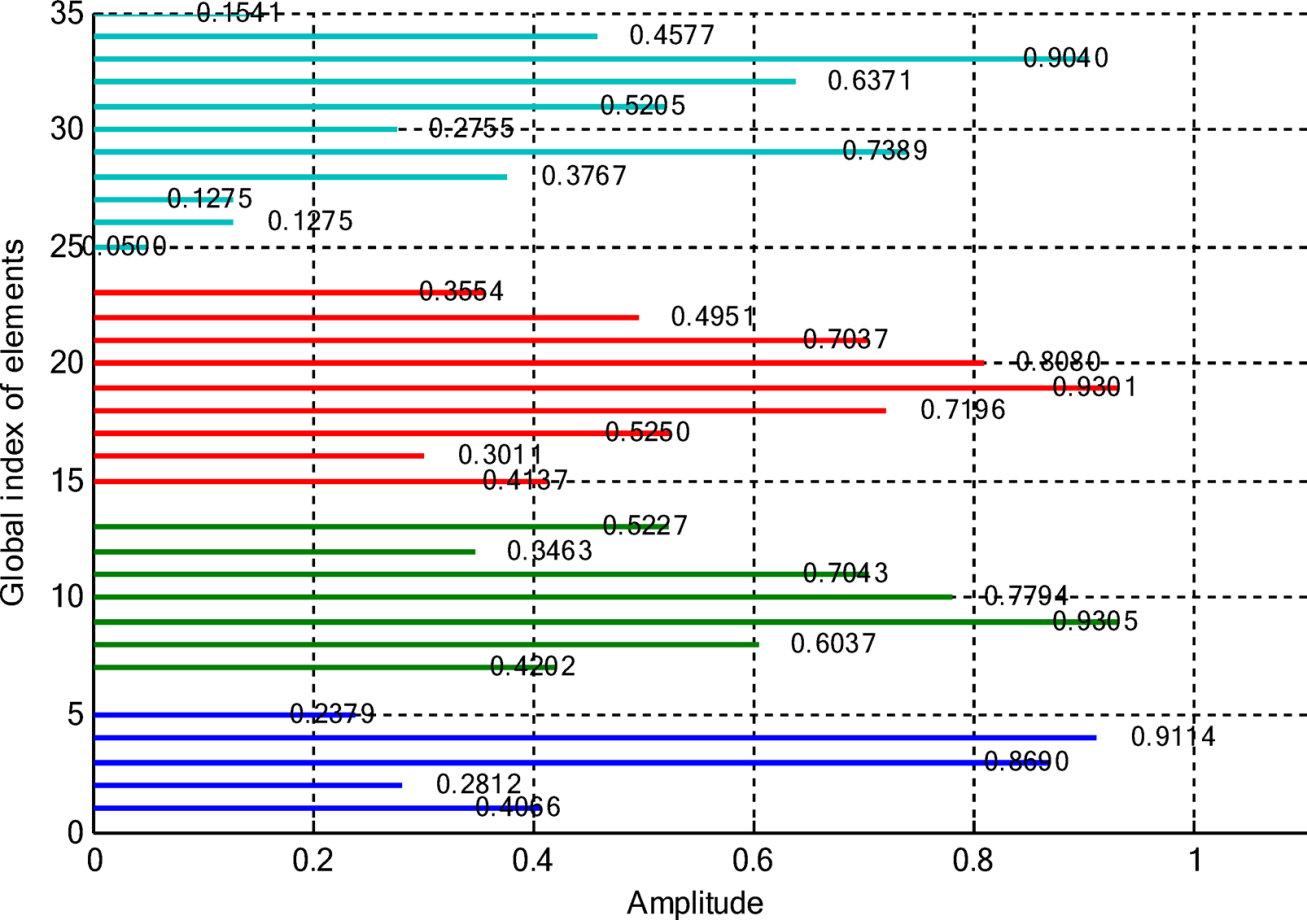
Optimized amplitude distribution for a CCAA configuration with 5, 7, 9, and 11 elements per ring using joint amplitude and ring radius optimization, including a central element.

**Fig. 21:**
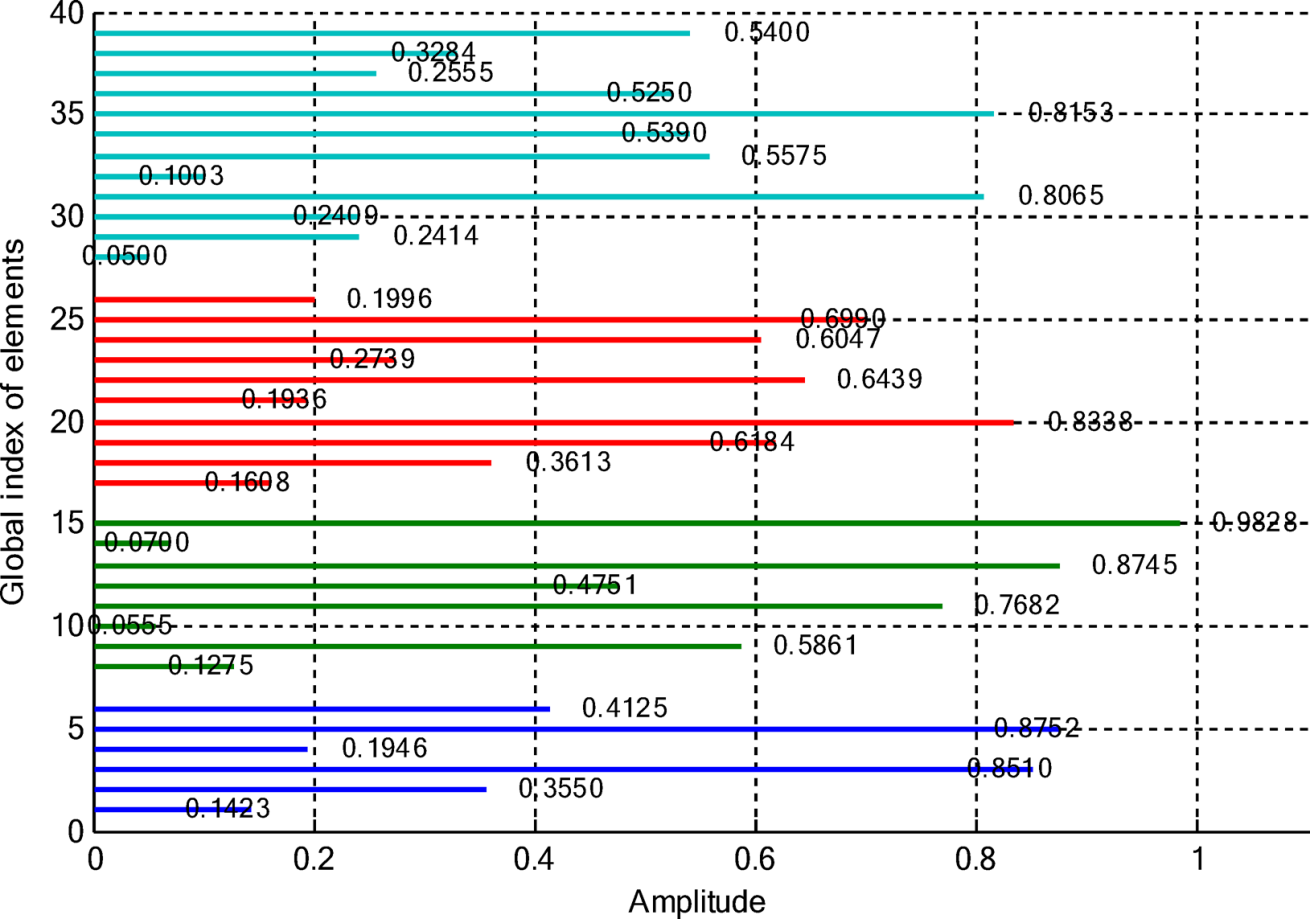
Amplitude distribution of CCAA with 8, 10, 12, and 14 elements using the amplitude and ring radii optimisation technique with a central element.

This case concerns the synthesis of CCAAs, including a central element optimized by the proposed hybrid algorithm capable of simultaneously optimizing excitation amplitudes and ring radii. Compared to arrangements composed of concentric rings only, the inclusion of the central element increases the design degrees of freedom so that radiation features such as main lobe shaping, SLL control, and symmetry can be more precisely controlled. However, this is at the cost of increasing the design problem complexity due to the possibility of stronger mutual coupling effects than in the previous cases. Figures 20 and 21 give the following amplitude distributions obtained with the proposed joint optimization strategy. Figure 20 refers to the arrangement with 5, 7, 9, and 11 elements per ring (33 elements in total), where optimized excitation amplitudes lie in the range from 0.0500 (element 20) to 0.9305 (element 7). In this instance, the algorithm can selectively turn on both inner- and outer-ring elements, such as element 1 having an amplitude of 0.4066 and element 16 having an amplitude of 0.9301, to effectively trade between high directivity and adequate sidelobe attenuation. By customizing the excitation distribution, the outcome is an improved sharpness of the main beam combined with reduced unwanted radiation in the sidelobe regions. The amplitude distribution illustrated in Fig. 21 corresponds to a denser configuration composed of 8, 10, 12, and 14 elements per ring (for a total amount of 45 elements), featuring wider dynamic ranges. Indeed, the optimized excitations range from 0.0500 to 0.9828 of element 22.

Notably, elements such as 5 (0.8752), 10 (0.7682), and 40 (0.8909) show high excitations, which support a more dispersed and finer beamforming strategy. This radiation pattern enhances radiation control due to the ability to manipulate both main lobe directionality and sidelobe suppression with great accuracy across the array structure. These results verify that the proposed hybrid PSO-FPA algorithm is effective in adaptively shaping the excitation profile for the reduction of sidelobe levels and improvement of beam precision. As well, the denser element arrangement assures higher spatial resolution, which helps control the overall radiation performance. Table 11 presents the optimized configurations of two CCAAs, each having a center element and synthesized using the developed hybrid optimization technique. The first array has four concentric rings, having 5 elements on the innermost ring, followed by 7, 9, and 11 elements on the subsequent rings.

**Table 11:** Array configuration, radius, SLL, and execution time for optimized CCAA using the suggested algorithm for case IV (with a central element).

Configuration	Ring	Radius	SLL (dB)	Execution time (Sec)
	5	1.1544	−45.30	10.62
	7	1.3870		
	9	1.6197		
	11	1.8496		
	8	0.7857	−44.75	12.19
	10	1.2612		
	12	1.5723		
	14	1.8834		

**Fig. 22:**
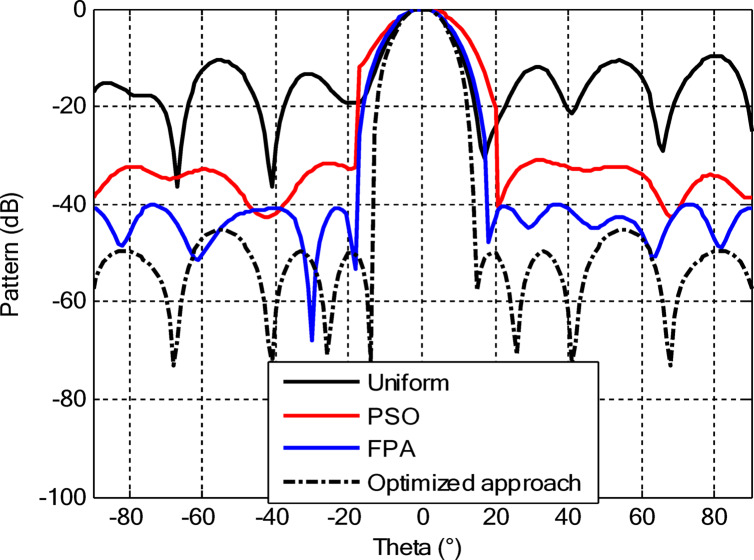
Beam patterns for Set N°1 (5, 7, 9, 11) optimized CCAA using amplitude and ring radius optimization technique, achieved by different algorithms (with a central element).

The optimized radii for this configuration are 1.1544, 1.3870, 1.6197, and 1.8496, respectively. In the second array, there is a denser arrangement of 8, 10, 12, and 14 elements in its four rings, with optimized radii of 0.7857, 1.2612, 1.5723, and 1.8834. These radii have been carefully computed so that elements are spatially distributed appropriately for better radiation while suppressing the SLLs with a focused main lobe. Beam patterns corresponding to the amplitude and ring radii optimized CCAA configurations, both having a central element, are depicted in Figs. 22 and 24. Their performance is compared for four excitation methods: uniform excitation, PSO, FPA, and hybrid optimization. Figure 22 corresponds to the 5, 7, 9, and 11 configurations. The hybrid approach has considerably improved the sidelobe suppression with an SLL of − 45.30 dB, which is much better than PSO (− 30.14 dB), FPA (− 34.62 dB), and the highest SLL due to uniform excitation. The main lobe is also sharper and symmetric, which indicates better directivity.

**Fig. 23:**
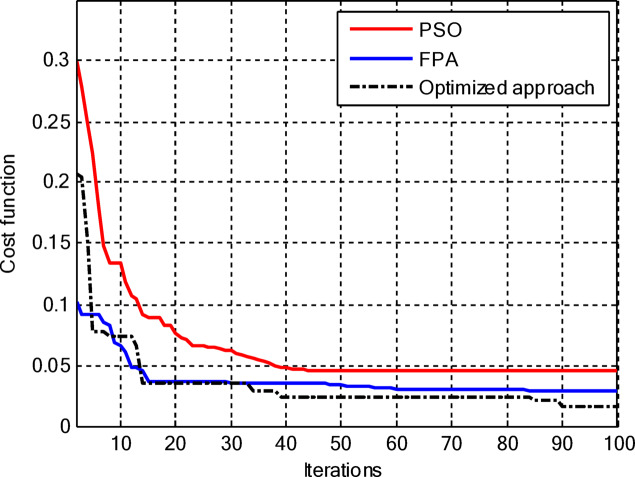
Convergence curve of CCAA for Case IV (Set I).

Figure 23 confirms the hybrid PSO–FPA’s superiority in amplitude and ring radius synthesis of CCAAs with a central element, reaching the lowest final cost (~ 0.05) compared to PSO (~ 0.12 plateau) and FPA (~ 0.08 stagnation). PSO delivers aggressive early convergence but prematurely flattens after ~ 15 iterations; FPA exhibits balanced but limited descent, while the hybrid sustains progressive refinement via integrated velocity updates and pollination mechanisms for optimal joint optimisation.

**Fig. 24:**
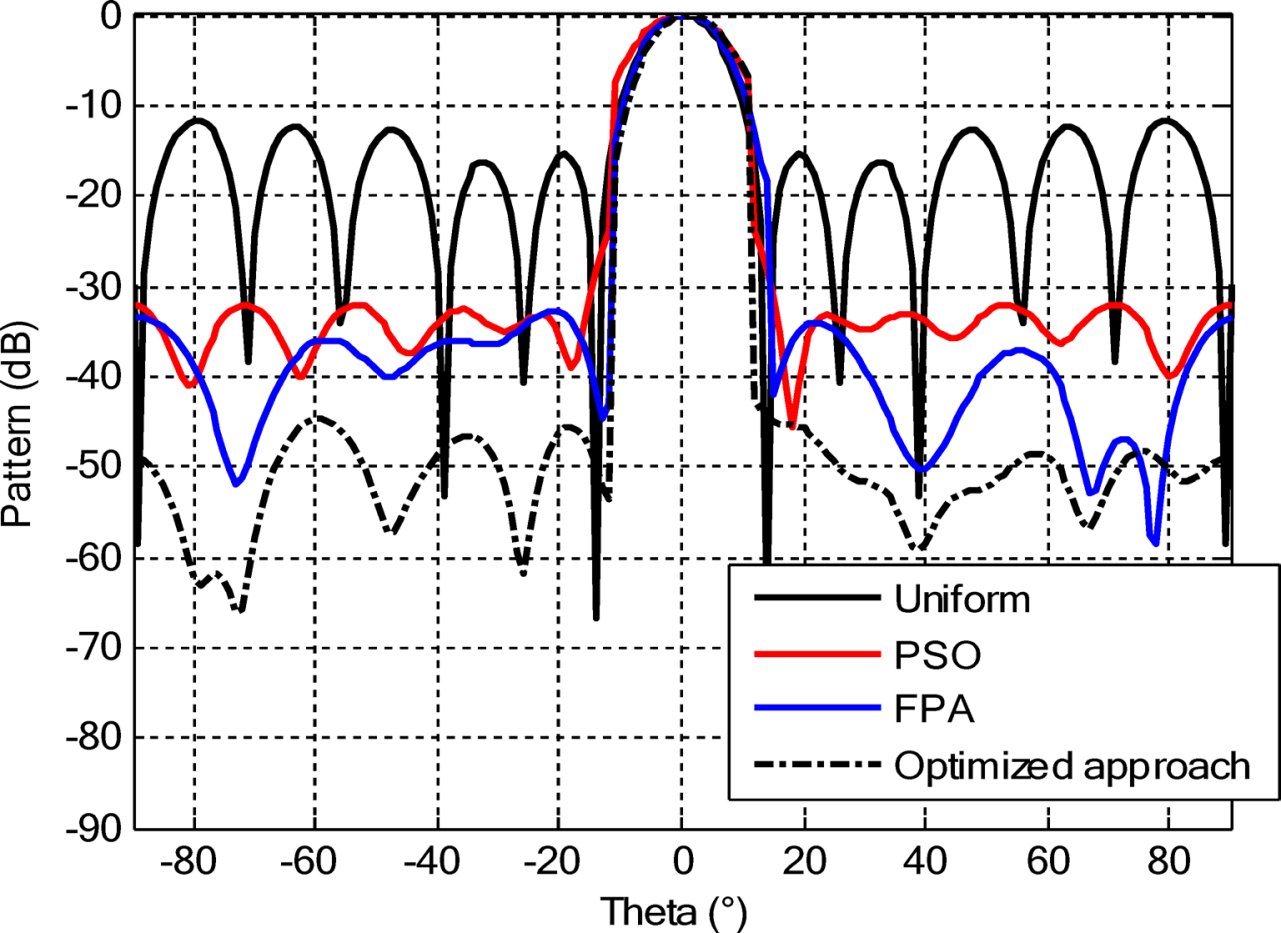
Beam patterns for Set N°2 (8, 10, 12, 14) optimized CCAA using amplitude and ring radii optimization achieved by different algorithms (with a central element).

Figure 24 presents the results of an even denser configuration (8, 10, 12, and 14 elements), where the hybrid technique outperforms the other techniques again, resulting in an SLL of −44.75 dB. By contrast, FPA and PSO exhibit higher sidelobe levels. The achieved radiation pattern in the case of this latter configuration is not only narrower but also more stable over the angular range. These benefits stem from the increased element density, while the joint amplitude and radius optimization leverage this higher density to obtain superior performance. This proves that the existence of a central element, together with optimized amplitude and ring radii parameters, considerably improves the beamforming performance and further verifies the performance of the introduced optimization strategy for high-precision, high-performance antenna array designs.

**Fig. 25:**
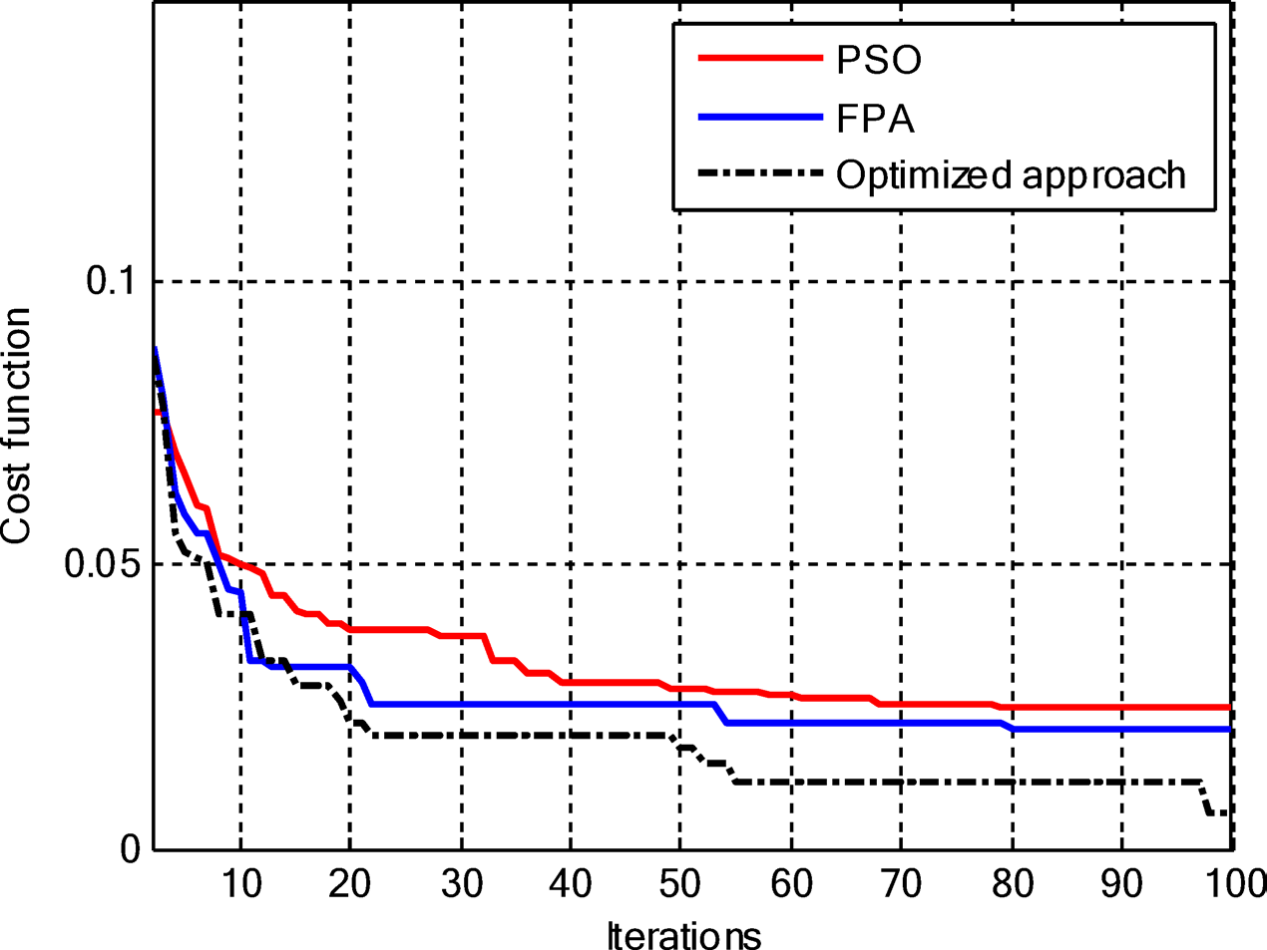
Convergence curve of CCAA for Case IV (Set II).

Figure 25 reveals that for the final CCAA configuration in amplitude and ring radius synthesis with the central element, the hybrid PSO–FPA attains the lowest cost plateau (~ 0.02), significantly outperforming PSO (~ 0.05 stagnation) and FPA (~ 0.03 plateau). All algorithms converge rapidly by iteration 30, but the hybrid’s sustained refinement through embedded PSO velocity in FPA phases enables superior final optimization in this most complex joint parameter space.

The results for Case IV (Table 12) conclusively reinforce the established superiority of the hybrid PSO-FPA algorithm. It consistently achieves the optimal sidelobe levels, approximately − 45 dB, while maintaining the lowest computational cost. This represents a decisive dual advantage: the algorithm is 13–29% faster than the benchmark methods while simultaneously improving sidelobe suppression by a significant 5 to 13 dB. The remarkable consistency of this performance—delivering both the best radiation pattern and the fastest convergence across all four comprehensive test cases—provides robust, empirical validation of the proposed method’s efficiency and reliability for synthesizing high-directivity, ultra-low sidelobe antenna arrays.

**Table 12:** CPU comparison of algorithms vs. SLL metrics for case IV.

Case No.	Set No.	Algorithm	CPU Time (s)	SLL (dB)
IV	I	PSO	12.33	−31.27
		FPA	14.56	−40.25
		Proposed method	10.62	−45.30
	II	PSO	14.09	−32.21
		FPA	17.22	−32.87
		Proposed method	12.19	−44.75

## Comparative study

**Fig. 26:**
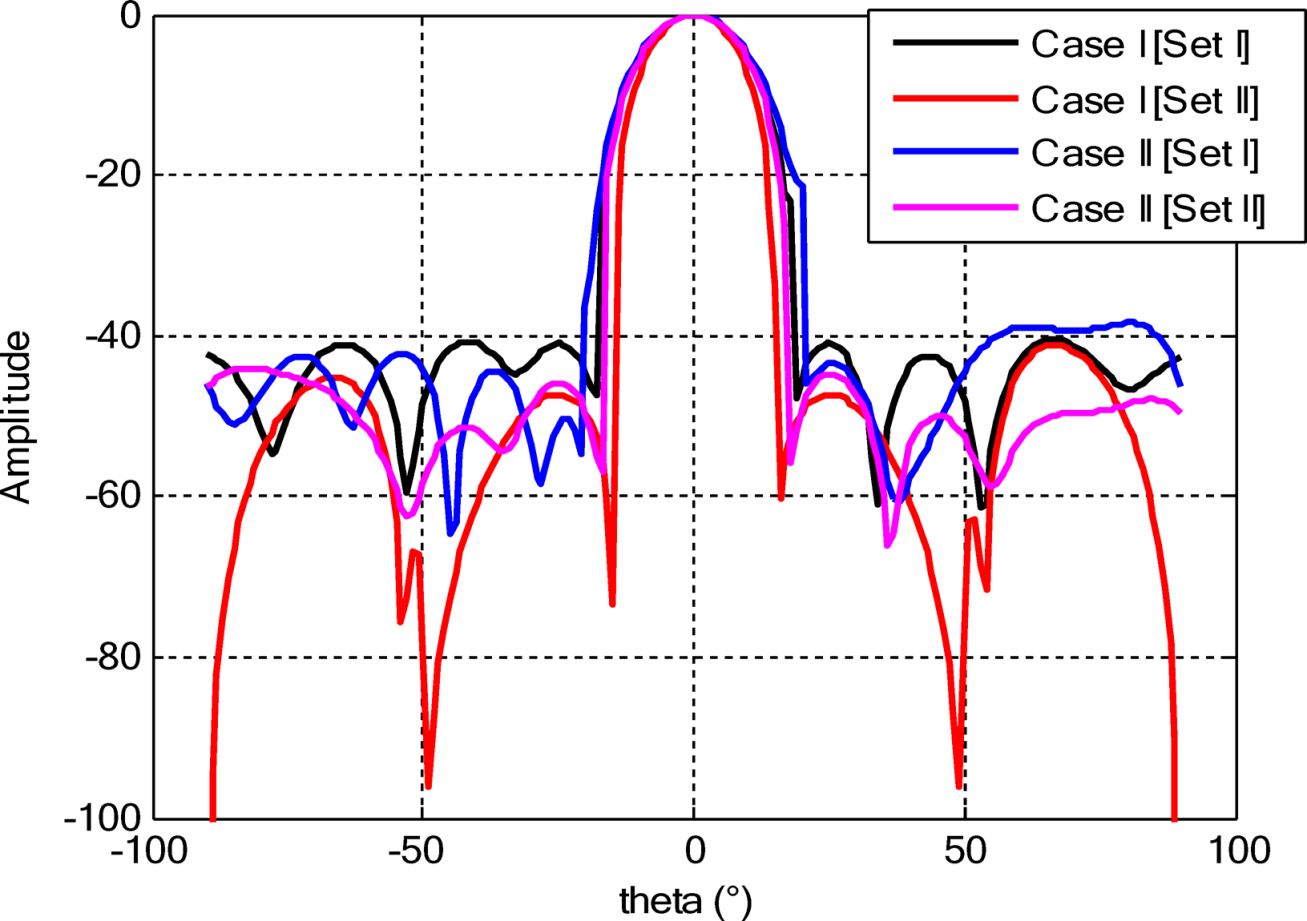
Beam patterns’ comparison for amplitude-only optimized CCAA without and with a central element achieved by the suggested algorithm.

**Fig. 27:**
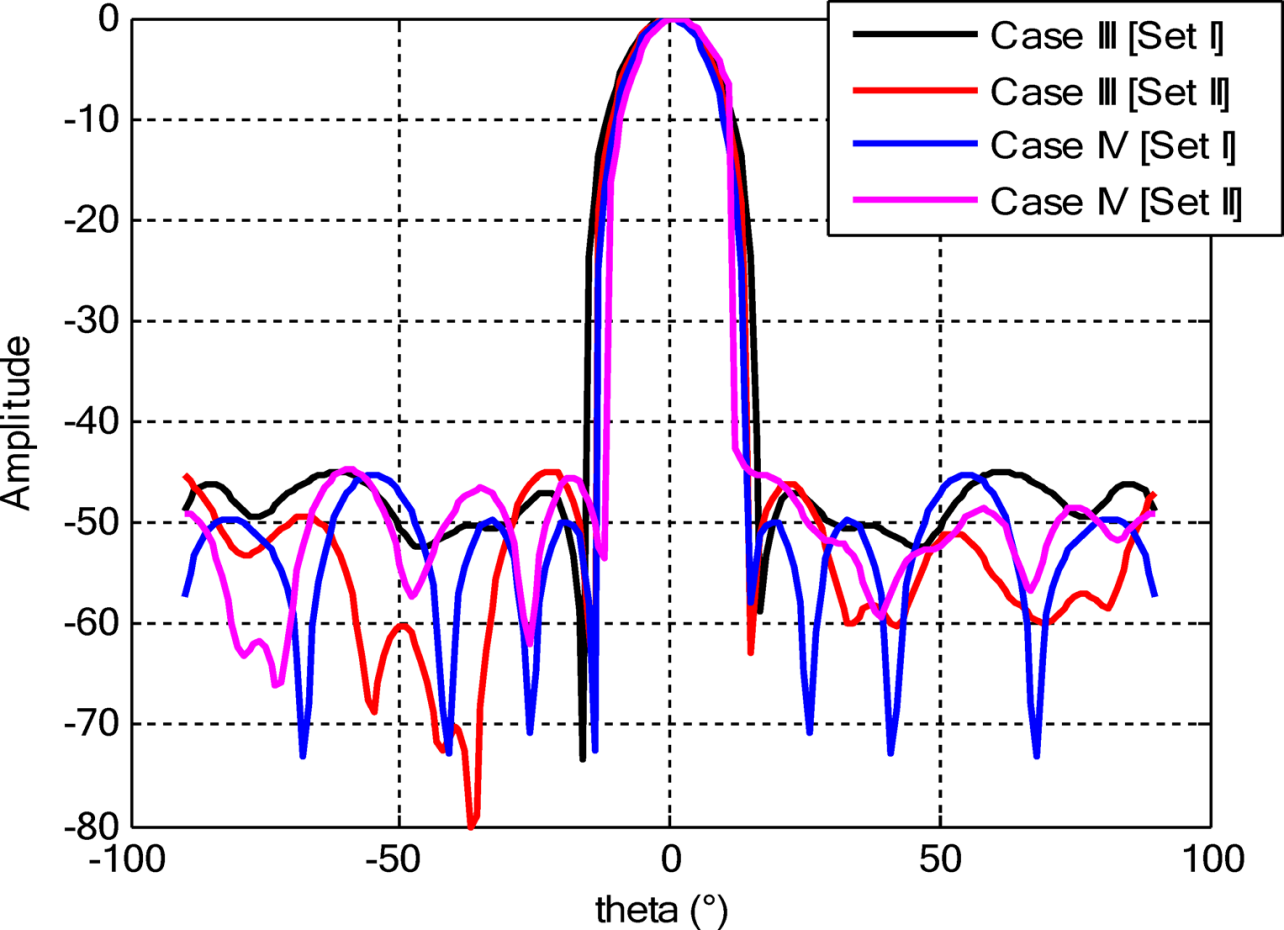
Beam patterns’ comparison for amplitudes and radii optimized CCAA without and with a central element achieved by the suggested algorithm.

Figures 26 and 27 present the detailed comparison of the radiation pattern results obtained by the proposed algorithm for the two different optimization scenarios: (i) optimization of excitation amplitudes alone, and (ii) combined optimization of excitation amplitudes together with ring radii. Figure 26 presents the computed AFs for the CCAA for two configuration sets (Set I and Set II) under two different cases, namely, without the central element (Case I) and with the central element, respectively (Case II). The analysis shows a marked improvement in the radiation characteristics when the central element is present. In fact, even for amplitude-only optimization, the central element significantly reduces the sidelobe levels and allows a more focused energy concentration within the main lobe. This corroborates that the central element is effective in enhancing the constructive interference along the main beam axis while suppressing spurious radiation in off-axis directions. Figure 27 shows the AFs derived from joint optimization of amplitudes and ring radii for configurations without (Case III) and with (Case IV) the central element. The integrated optimization strategy accentuates the performance of the array even further, particularly in terms of symmetry of the beams, narrowing of the main lobe, and attenuation of sidelobes. Note that Case IV, including the central element, attains the highest directivity and the cleanest radiation profile. This implies that the optimized excitations and spatial distribution of the elements provide a synergistic effect in pattern refinement. Collectively, these observations validate the cardinal importance of the central element in CCAA configurations. Its presence not only enhances directional gain but also contributes to better sidelobe control. Moreover, the dual optimization approach further amplifies the benefits and thus points out the clear design path toward high-performance antenna arrays with a tailored beam pattern.

From a theoretical point of view, this central element acts like an electromagnetic anchor that stabilizes the structure of radiation and allows for finer control of the constructive and destructive interference phenomena. This freedom provided due to its presence is especially welcome in concentric geometries where radial symmetry can be exploited to enhance beamforming. Numerical results from all case studies establish the necessity of accurate tuning in both amplitude and spatial parameters, especially in the case of arrays with a centrally placed radiator, to achieve the best possible performance of the array. Therefore, careful attention must be paid to the design, excitation, and placement of this central radiator to fully exploit its potential in state-of-the-art array synthesis.

For this considerations, real-world transition in CCAA optimisation introduces significant complexity, requiring sophisticated cost functions that incorporate multi-objective terms—such as balancing sidelobe levels (SLL), bandwidth, and radiation efficiency—alongside nonlinear constraints like minimum element spacing or excitation dynamic range limits and direct integration with full-wave electromagnetic (EM) solvers for realistic performance prediction, accounting for mutual coupling and fabrication effects.

In these demanding scenarios, the PSO–FPA hybrid demonstrates clear superiority over conventional gradient-based methods, which falter in discontinuous, noisy, non-convex, and multimodal landscapes typical of antenna design. As a derivative-free, population-based approach, PSO–FPA exhibits inherent robustness: FPA’s Lévy flight-based global pollination ensures escape from local optima through broad exploration, while PSO’s velocity-updated social and cognitive components enable efficient local refinement in promising regions, making the hybrid exceptionally adept at navigating intricate, high-dimensional design spaces.

The algorithm’s black-box nature provides unparalleled flexibility, allowing seamless incorporation of any computable metric into a unified aggregate cost function—whether mutual coupling extracted from S-parameter matrices, pattern distortions from radome/housing effects, or feeding network efficiency—without needing differentiable or closed-form analytical expressions, which are practically unattainable for such coupled physical phenomena.

Demonstrated through convergence plots, the hybrid achieves optimal exploration-exploitation synergy, outperforming standalone PSO (premature stagnation) and FPA (limited refinement). Rapid initial descent was followed by meticulous final tuning—critical for complex costs where suboptimal convergence risks are high.

Although population methods demand many costly evaluations (e.g., CPU-intensive EM simulations), the hybrid’s empirically faster convergence—evidenced by reduced CPU times across all configurations—minimises required iterations, directly offsetting this expense and establishing PSO–FPA as strategically superior for advanced, constraint-heavy antenna synthesis bridging simulation and practical deployment.

## Conclusions

This study developed an efficient hybrid metaheuristic optimization algorithm that preserves the global exploratory capability of the FPA and adaptive convergence and local fine-tuning ability of PSO for the synthesis of the CCAAs. The novel PSO–FPA algorithm achieves a good balance between exploration and exploitation through the use of FPA’s global pollination operation and inertia-weighted velocity update of PSO. This equilibrium makes convergence velocity quicker, the quality of solutions improved, and radiation behaviour control more efficient. The algorithm was tested in different design cases, including amplitude-only optimization and simultaneous optimization of amplitudes and ring radii with and without a central element. Results validated that the hybrid algorithm achieved a minimum SLL of − 45.01 dB, an improvement of over 40% when compared to classical FPA and PSO methods, and improved directivity up to 13.14 dB, signifying more focused main beams and improved symmetry. Including the central element contributed towards improved beam shaping and energy concentration within less than 12 s of computational time per configuration. The simulation results confirm that the proposed hybrid algorithm is scalable, convergent, and of high accuracy, and it could be regarded as an appropriate tool for antenna design in future wireless communication, radar, and remote sensing systems. Future works will include the experimental implementation, mutual coupling investigation, and real-time adaptation to dynamic environments, where the integration of reinforcement learning and ML techniques will also be explored to enable parameter tuning through automated process adjustment and intelligent decision-making.

The simulation results clearly show that the proposed PSO-FPA algorithm is better at CCAA synthesis than other methods. However, putting these optimised designs into real-world arrays will require overcoming a number of major technical challenges. This encompasses alleviating the impacts of manufacturing tolerances and component discrepancies, counteracting significant mutual coupling among closely positioned elements, and implementing the intricate beamforming networks necessary for non-uniform excitations—all of which can diminish the sidelobe levels and directivity attained. Moreover, for radar applications necessitating adaptive reconfiguration, the algorithm’s computational efficiency must be assessed on embedded hardware for real-time functionality, and its overall performance must be demonstrated by accurate measurements in an anechoic chamber. Thus, forthcoming research will concentrate on incorporating full-wave electromagnetic analysis for coupling-aware synthesis, formulating resilient design methodologies to counteract imperfections, and investigating the implementation trade-offs for practical beamforming networks, thereby reconciling the disparity between high-performance simulation and dependable physical deployment for advanced radar systems.

## Data Availability

The datasets used and/or analyzed during the current study available from the corresponding author on reasonable request.

## Abbreviations

FPA: Flower Pollination Algorithm
PSO: Particle Swarm Optimization
CCAAs: Concentric Circular Antenna Arrays
SLL: Side Lobe Levels
ABC: Artificial Bee Colony
WOA: Whale Optimization Algorithm
GA: Genetic Algorithm
BBO: Biogeography-Based Optimization
FA: Firefly Algorithm
SOS: Symbiotic Organisms Search
AF: Array Factor
CF: Cost Function
BSA: Backtracking Search Optimisation
PO: Political Optimiser
ML: Machine Learning
